# Forest canopy-cover composition and landscape influence on bryophyte communities in *Nothofagus* forests of southern Patagonia

**DOI:** 10.1371/journal.pone.0232922

**Published:** 2020-11-24

**Authors:** Mónica D. R. Toro Manríquez, Víctor Ardiles, Álvaro Promis, Alejandro Huertas Herrera, Rosina Soler, María Vanessa Lencinas, Guillermo Martínez Pastur

**Affiliations:** 1 Laboratorio de Recursos Agroforestales, Centro Austral de Investigaciones Científicas (CADIC-CONICET), Ushuaia, Tierra del Fuego, Argentina; 2 Área de Botánica, Museo Nacional de Historia Natural, Quinta Normal, Santiago, Chile; 3 Facultad de Ciencias Forestales y de la Conservación de la Naturaleza, Universidad de Chile, La Pintana, Santiago, Chile; Oregon State University, UNITED STATES

## Abstract

Bryophytes (liverworts, mosses and hornworts) are one of the most diverse plant groups worldwide but one of the least studied in temperate forests from an ecological perspective. In comparison to vascular plants, bryophytes have a broader distribution and a longer altitudinal gradient, and their influence on the landscape is poorly understood. The objective was to evaluate environmental drivers that can influence bryophyte cover, richness, diversity, and nestedness in different forest canopy compositions in two typical landscapes across the natural distribution of bryophytes in Tierra del Fuego (Argentina). Three natural *Nothofagus* forest types (pure deciduous, pure evergreen, and mixed deciduous-evergreen) in two landscapes (coasts < 100 m.a.s.l. and mountains > 400 m.a.s.l.) were selected (N = 60 plots). In each plot, we established one transect (10 m length) to measure bryophyte cover (point-intercept method). Data were evaluated using generalized linear mixed models and multivariate analyses. The studied environmental drivers were mainly explained by the microclimate, with higher effective annual precipitation and relative air humidity in the coastal forests and higher soil moisture in the mountain forests. Greater liverwort richness was found in evergreen forests at the mountain (9 species) than at the coastal, while mosses showed higher richness in mixed deciduous-evergreen forests at the coastal (11 species) than at the mountain. However, the expected richness according to the rarefaction/extrapolation curves suggested that it is possible to record additional species, except for liverworts in pure deciduous forests on the coasts. Similarities and differences among the studied forest types and among plots of the same forest type and landscape were detected. These differences in the studied indexes (similarity that varied between 0 and 1) ranged from 0.09–0.48 for liverworts and 0.05–0.65 for mosses. Moreover, these results indicated that pure evergreen and mixed deciduous-evergreen forests presented higher moss cover (10.7% and 10.0%, respectively), mainly in the mountains than on the coast. These outputs highlight the need to explore differences at greater altitudinal ranges to achieve sustainability objectives conservation planning for bryophytes in southernmost forests.

## Introduction

Bryophytes (liverworts, mosses and hornworts) are distributed globally, occur in all terrestrial ecosystems [1] and are one of the earliest and most diversified groups of land plants, constituting one of the most important components of vegetation diversity [2]. Many species show wide transcontinental ranges, where the occurrence of bryophyte species may be a vestige of the mossflora prior to the rupture of Laurasia and Gondwana [3]. Bryophytes have a broader distribution and a longer altitudinal gradient than vascular plants (e.g., woody plants, ferns) [4]. In forests, bryophytes play crucial ecological functions as primary producers (e.g., carbon and nutrients cycle, water balance) [5–8]. However, bryophytes are smaller than vascular plants and are often ignored in ecological studies [1, 9] and are usually only included during floristic surveys [1]. Currently, forest research on the diversity and distribution of bryophytes has increased in recent years due to their role in ecosystem functioning and their potential influence on climate change [4, 10, 11].

Liverwort and moss cover and richness are related to canopy cover, microclimate and other site conditions in stands (e.g., forest structure, air humidity, light intensity, substrate types, soil moisture, pH, understory composition) [6, 12–15]. Hence, exogenous factors may influence their narrow ecological requirements (e.g., microclimate and habitat modifications due to human uses) and consequently affect local biodiversity [14–16]. In addition, different landscapes can lead to differences in soil moisture, edaphic properties and other forest floor characteristics and can explain the main variation in patterns of plant cover, diversity and composition [17, 18]. Moreover, it is well known that temperature, precipitation and air humidity differ along elevation gradients and can influence bryophyte richness patterns, which can also be modified by the elevation [1, 2, 6]. Therefore, to ensure the conservation of bryophytes, it is necessary to identify the major drivers that influence liverwort and moss distributions [19, 20].

Southern Patagonia contains one of the last well-preserved wilderness regions on the planet [21], where *Nothofagus* trees represent the southernmost forests in the world [22]. These native forests occupy an extensive area and are dominated by deciduous *N*. *antarctica* (G. Forst.) Oerst., and *N*. *pumilio* (Poepp. et. Endl) Krasser, and evergreen *N*. *betuloides* (Mirb.) Oerst. These tree species grow naturally across the landscape, and some of them (*N*. *pumilio* and *N*. *betuloides*) can form mixed deciduous-evergreen forests [23, 24]. The understory is mainly composed of forbs, grasses and several woody shrub species, where pure deciduous forests and mixed forests present similar species compositions [25]. Here, bryophytes have not been included in most ecological studies. Previous studies on the community ecology of liverworts and mosses in *Nothofagus* forests pointed to the high variability in the ground-bryophyte communities related to forest structure heterogeneity and different substrate conditions [26, 27]. However, bryophytes and their environmental drivers (e.g., forest types and landscapes) are poorly studied.

The objective of this study was to evaluate the main drivers that influence bryophyte richness, cover and diversity according to the forest canopy-layer composition (pure deciduous, pure evergreen and mixed deciduous-evergreen forests) in two typical landscapes (coasts and mountains) in Tierra del Fuego (Argentina) and to contribute to explaining how the bryophyte communities vary according to the studied environmental drivers. We hypothesize that: (i) the main drivers that influence the cover, richness and diversity of bryophytes are forest structure, microclimate, and forest floor characteristics, which differ among the different forest types (e.g., light-sensitivity of bryophytes in deciduous forests) and between coasts and mountains (e.g., soil moisture); (ii) the cover and richness of liverworts and mosses are higher in evergreen forests than in deciduous forests, while mixed forests presented intermediate values, indicating the differences between coasts and mountains, because mountains have higher cover and richness the coasts; (iii) differences and similarities between pure and mixed forests occur in different landscapes (coasts or mountains) where the diversity is higher in mountains and pure evergreen forests than on the coast; and (iv) in comparison to mixed forests, pure forests have a higher number of exclusive plant species, which can be considered indicator species for the different forest types (e.g., deciduous or evergreen) and landscapes (e.g., coasts or mountains). This study might contribute to identifying strategies and opportunities for the conservation of bryophyte species and to generating basic knowledge about the environmental drivers that influence different temperate forest landscapes.

## Methods

### Study area

Research and sampling permits were granted for coastal locations at Tierra del Fuego National Park by the Administración de Parques Nacionales (DRPA/19/2014-2015) and mountains locations by the Secretary of Environment and Sustainable Development Tierra del Fuego (S.D.S. and A. N°0155/2014-2015).

**Fig 1:**
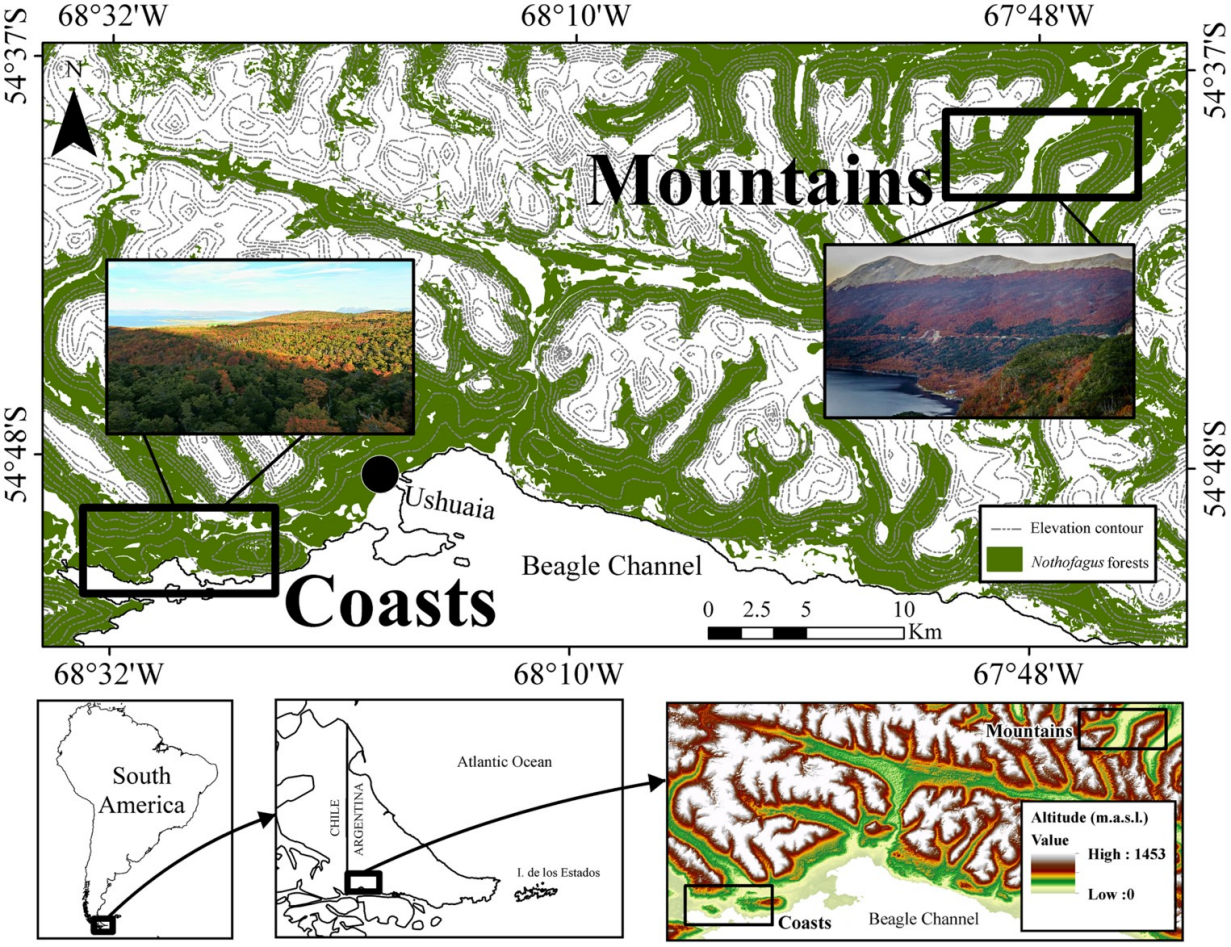
Locations of the study areas in southwestern Tierra del Fuego and altitude (m.a.s.l.). Rectangles indicate the sampled landscapes: (i) coasts of the Beagle Channel, and (ii) mountains in the inner island. *Nothofagus* forests appear in green, and photos next to the rectangles are examples of forest landscapes (credits by A. Huertas Herrera and J.M. Cellini).

This study was conducted in old-growth *Nothofagus* forests (>250 years-old) located in the southwestern Tierra del Fuego Island, Argentina (Fig 1), that has not had harvesting or cattle grazing during occur the last 60 years. Three *Nothofagus* forest types were considered, according to their canopy-layer composition: (i) Np = pure deciduous forests (with > 80% of *N*. *pumilio* canopy cover); (ii) Nb = pure evergreen forests (with > 80% *N*. *betuloides* canopy cover); and (iii) M = mixed deciduous—evergreen forests (with a similar proportion of *N*. *pumilio* and *N*. *betuloides* species in the overstory canopy) (see more details on overstory canopy characterization in Toro Manríquez et al. [23, 24] and Mestre et al. [25]). Similarly, two contrasting landscapes were selected, where these three forest types naturally occur: (i) marine coasts close to the Beagle Channel and (ii) mountainous areas toward the inner island. The coastal landscapes are within Tierra del Fuego National Park, where the altitude varies between 50 and 100 m.a.s.l., the mean annual air temperature is 4.3°C, and the annual precipitation is 756 mm yr^-1^ with abundant snowfall during winter. The forests in the mountain landscape are in Garibaldi Pass (450 m.a.s.l.), within the Andes Mountain range, where the annual temperature is 3.1°C, and the annual precipitation recorded is 788 mm yr^-1^ [28]. In general, the main soil type in both forest types on these coasts and in the mountains is textured loam, with large granular structures, low usable water capacity and moderate to slow internal and external drainage with a thick organic soil layer [29]. However, coastal and mountainous soils showed some differences; for example, canopy composition in these forests has an impact on soil properties, but most soil properties are strongly influenced by the landscape (e.g., in comparison to coastal soils, mountainous soils are wetter and richer in N and P) and, to a lesser extent, by the proportion of deciduous and evergreen canopies [24]. In addition, litterfall (quantity and quality), which determines nutrient cycling in soils, also differs among forest types and landscapes [24].

### Sampling design and measurements

#### Forest structure, microclimate and forest floor characteristics

The study design consisted of 10 plots per forest type and landscape distributed in an area of 300 ha on the coast and 100 ha in the mountains (3 forest types × 2 landscapes × 10 replicas = 60 plots). The plots were at least 50 m with a maximum of 1 km apart in the same sampling area. The forest structure was characterized by angle-count sampling plots, following the method proposed by Bitterlich [30]. We used a laser Criterion RD-1000 (Laser Technology, USA) with a variable basal area factor k = 6–7 to calculate the basal area of the stands (m^2^ ha^-1^). Additionally, dominant height (m) was measured using a TruPulse-200 laser clinometer and distance rangefinder (Laser Technology, USA) by averaging the height of the three tallest trees per plot. In addition, the species and diameter at breast height (cm) were recorded for each tree. At the center of each plot, hemispherical photos of the canopy were taken. We used an 8 mm fish-eye lens Ex-AF4 (Sigma, Japan) mounted on a 35 mm digital camera D50 (Nikon, Japan) with a tripod and level, which was oriented to the north. The camera was set 1 m above the forest floor, which was enough distance to avoid registering understory or shrub cover in these austral forests and only capture tree canopy cover. The program Gap Light Analyzer v2.0 [31] was used to calculate canopy cover (%), relative leaf area index and transmitted total solar radiation (%) (ratio of direct plus diffuse radiation transmitted through the canopy, and the total radiation incident on a horizontal surface located above forest canopy). For more details on the methodology see Martínez Pastur et al. [32]. These data were obtained by taking one photo per plot each month during the growing season (October to March) to avoid the influence of direct sunlight.

We used two data loggers (model H8) per forest type and landscape (HOBO, USA) to characterize the microclimate in the studied forests (2 data loggers × 3 forest types × 2 landscapes, N = 12), measuring soil and air temperature (°C) and relative air humidity (%). Finally, to measure the effective annual precipitation (PP, mm yr^-1^) through the canopy, twelve Watermark Watchdog 425 data loggers (Spectrum, USA) were installed. All microclimatic variables were measured between October 2014 and March 2015.

Soil resistance to penetration and soil moisture were evaluated by five measurements per plot (5 measurements per plot N = 300 measurements) during January 2015. The soil resistance to penetration (N cm^-2^) was determined by a manual penetrometer (Eijkelkamp Agrisearch Equipment, Netherlands) up to 30 cm deep, while the soil moisture (%) was determined with an MP406 moisture probe (ICT, Australia) in the first 10 cm of the soil layer. At the same time, five mineral soil sub-samples from each plot (5 sub-samples per plot, N = 300 subsamples) were taken at depths of 0–20 cm with a soil auger. The sub-samples were dried in an oven at 70°C, ground in an analytical mill type (Cole-Parmer USA) and then sieved (2 mm). The soil pH was also determined at the Laboratorio de Recursos Agroforestales (CADIC) using a pH meter (Orion, USA). The subsamples were pooled to form a single sample per sampling plot (60 measurements in total). The slope (%) was calculated for each plot with a clinometer (Suunto, Finland).

#### Bryophyte composition

We established one linear transect (10 m length) in each plot (10 transects × 3 forest types × 2 landscapes = 60 transects). For the measurements, we employed the point-intercept method [33] completing 50 intercept points per transect (one observation every 20 cm) to record bryophyte species considering the two main groups: mosses and liverworts. This method allowed us to record each plant species intercepted (one or more species at different vegetation strata) at the same point. The total number of interceptions for all species over the 50 points provides an estimation of the total cover [25, 34]. For species identification, a 0.5 cm^2^ sample was collected at each point. With these data, we calculated the species richness (moss and liverwort richness) and cover (moss and liverwort cover) for each transect. On the same transect, we also recorded other forest floor cover characteristics, such as bare soil (%) which encompassed litter cover or soil without vegetation; woody debris cover (%), which included branches and trunks ˃ 3 cm diameter; vascular plants (%) which included ferns, monocots and dicots; and lichen cover (%). Fieldwork was conducted during summer (2015) which is the most appropriate period for vegetation surveys in *Nothofagus* forests at this latitude (within the growing season) [26].

For species determinations, we followed Ardiles et al. [35] and Ardiles and Peñaloza [36]. We carried out microscopic analyses of structural details (e.g., specific parts of the gametophytes and sporophytes), confirming and/or complementing the taxonomic information. These determination were conducted at the Botanical Area of the Museo Nacional de Historia Natural (Santiago, Chile). We used the nomenclature proposed by Müller [37] for moss species and that by Hässel de Menéndez and Rubies [38] for liverworts. To establish the global distribution pattern (GDP) analyses of each species, we followed the proposal of León et al. [39], which is essentially an adaptation of the patterns proposed by Seki [40] and Villagrán et al. [41]. According to these approaches, species can be classified as: (i) disjunct with South America, South Africa and Europe; (ii) endemic; (iii) pantropical-type *Podocarpus*; (iv) austral-Antarctic; (v) cosmopolitan; and (vi) bipolar. We also analyzed the occurrence frequency (%) of the studied taxonomic groups (mosses and liverworts) (S1 Table). Finally, we determined the substrate where the specimens were growing, which were registered at each sampling point in the transects, and the substrates were litter, decaying wood, bare soil, stones, and epiphytic on branches and/or bark on the forest floor.

### Data analyses

We tested the effects of forest type (pure deciduous, mixed deciduous-evergreen, pure evergreen forests) and landscape (coasts, mountains) on forest structure (basal area, dominant height, diameter at breast height, canopy cover), microclimate (transmitted total solar radiation, soil moisture, effective annual precipitation, soil temperature, air temperature, relative air humidity), soil and forest floor characteristics (slope, soil pH, soil resistance to penetration, and ground cover discriminated as bare soil, woody debris, vascular plants and lichens) and moss and liverwort cover, using generalized linear mixed models (GLMMs). Moreover, we used GLMMs to test the effects of tree species (*N*. *pumilio* and *N*. *betuloides*) and landscapes (coasts and mountains) on forest structure (basal area, dominant height, diameter at breast height) in mixed forests. The models with Gaussian distributions had the best fit all variables, except for effective annual precipitation, soil temperature, air temperature and relative air humidity, for which we used the Poisson distribution. Comparisons of the mean values were conducted by the LSD (Least Significant Difference) Fisher test (p < 0.05). Statistically significant interactions were plotted for better interpretation of the results. We used InfoStat software [42] to perform these analyses.

We analyzed differences in species richness and diversity for liverworts and mosses in each forest type and landscape using sample size-based approaches [43]. Species diversity curves were constructed with Hill numbers: species richness (q = 0), exponential Shannon entropy (Shannon’s diversity, q = 1), and inverse Simpson concentration (Simpson diversity, q = 2) for sample size-based rarefaction and extrapolation curves [43, 44]. Extrapolations were conducted from an incidence data matrix (Hill number of order = 0) [43]. The sample size corresponded to the sampling carried out by point interception (10 transects × 50 point-sampling = 500 observed sampling units in the reference for incidence data) for each forest type and landscapes. Sample-based rarefaction/extrapolations were calculated using iNEXT (iNterpolation and EXTrapolation) online [45]. All three Hill numbers were estimated as the mean of 100 replicate bootstrapping runs to estimate 95% confidence intervals. Extrapolations were extended to a sample size of 1000 for all sites. In the analyses, whenever the 95% confidence intervals did not overlap, the species number differed significantly at p < 0.05 [44]. Moreover, we calculated the incidence‐based Chao-Sørensen similarity index [46, 47] to determine differences among plots, both for forest types and landscapes, analyzing liverworts and mosses separately. This index, which ranges from 0 to 1, explicitly considers the relative incidence of both common and rare species and estimates the extent of shared species taking into account unseen shared species, based on the number of observed shared rare (singletons and doubletons, as well as unique and duplicate), species between two forest types and landscapes. The use of this index is recommended when study sites could be under sampled and can contain only a substantial fraction of the rare species [47]. The forest types and landscapes were ordered from highest to lowest according to the average value of similarity of the comparisons among the different forest types and landscapes, measuring each against each and including internal comparisons (similarity between plots of the same forest types and landscapes). We used the software EstimateS [48] for each pairwise evaluation, and we compared averages and standard deviations for each forest type and landscape.

For the nestedness evaluation of mosses and liverworts, we used nestedness measure based on the overlap and decreasing fills (NODFs), which are recommended based on their better response compared with that of other similar methods [49]. NODFs generate data that range from 0 (no nestedness) to 100 (perfect nestedness). To test the performance of our species assemblages against a constrained "fixed-fixed" model, we employed a null model (999 replicates) [50]. More details about this type of analysis can be found in Almeida-Neto et al. [49]. To separate the effects of nestedness, we evaluated the forest types by comparing the whole study site jointly, each forest type in each landscape separately, and each forest type by comparing the landscapes together. NODFs were calculated with NeD software [51].

Multivariate methods were also conducted (using the species cover data) as complementary analyses: (i) indicator species analysis (IndVal) for bryophyte species composition, comparing forest types and landscapes. The maximum value of the IndVal among groups (forest types and landscapes, separately) was evaluated to determine its statistical significance (p < 0.05) using a Monte Carlo permutation test (number of runs 4999) [52]. We followed the criteria used by Promis et al. [27], which consider an indicator species of nonvascular plants with an IndVal ≥ 25 and values of p < 0.05. (ii) Canonical correspondence analyses (CCAs) based on species cover data (mosses and liverworts) were used to test the relationships among bryophyte communities and the studied environmental drivers. First, Pearson correlation was used for the environmental drivers (p < 0.05), values between -1 and 1 (above 0.5 or below -0.5 indicates the existence of significant correlations) were assigned to them. Similarly, the Monte-Carlo method with 499 permutations was employed to test the significance of each axis in the CCAs. All multivariate analyses were performed using PC-ORD [53].

Complementarily, for the substrate analyses, we compared the treatments using contingency tables and the chi-square test, considering comparisons of forest types and landscapes: CNp = pure deciduous forests on the coasts, CM = mixed deciduous-evergreen forests on the coasts, CNb = pure evergreen forests on the coasts, MNp = pure deciduous forests in the mountains, MM = mixed deciduous-evergreen forests in the mountains, MNb = pure evergreen forests in the mountains. The chi-square test was conducted using SPSS Statistics version 25.0 [54].

## Results

A total of 27 bryophyte species were recorded: 56% were mosses (15 species belonging to the Bryophyta division) and 44% were liverworts (12 species belonging to the division Marchantiophyta and subclass Jurgemanniopsida). The global distribution pattern for the recorded species showed that mosses were austral- Antarctic (53%), pantropical-type *Podocarpus* (20%), cosmopolitan and bipolar (7% for each). The liverwort group mainly were: (i) endemic (58%) and (ii) disjunct with South America, South Africa and Europe (33%), while a small proportion was undetermined (8%). The most common bryophytes were the moss *Acrocladium auriculatum*, recorded mainly in the deciduous forests and mountain landscapes (48% mean occurrence frequency among the studied forest types and 32% between the landscapes), and the liverwort *Adelanthus lindbergianus*, mainly recorded in the evergreen forests of the mountains (32% mean occurrence frequency among forests types, and 21% between landscapes). The lowest occurrence frequencies (1.7% in average among the different forest types, and 1.1% in average between landscapes) were found in the coastal landscapes, where three species were mosses (*Bartamia mossmaniana* and *Hymenodontopsis mnioides* in mixed forests, and *Hennediella densifolia* in deciduous forests) and two species were liverworts (*Heteroscyphus intergrifolius* in deciduous forests and *Lophozia sp*. in mixed forests) (S1 Table).

### Forest structure, forest-floor cover and stand microclimate

The forest structure, except for canopy cover (%), showed significant differences among the different forest types and landscapes (Table 1). The basal area was higher in pure evergreen forests than in mixed deciduous-evergreen forests and pure deciduous forests (F = 7.10, p = 0.002). Similarly, the basal area was higher in the mountains than on the coasts (F = 5.80, p = 0.020). Dominant height (F = 12.31, p < 0.001) and diameter at breast height (F = 33.35, p < 0.001) presented significant differences along the studied gradient: pure deciduous forests > mixed deciduous-evergreen forests > pure evergreen forests. The RLAI was higher and similar in pure deciduous and mixed deciduous-evergreen forests than in pure evergreen forests (F = 10.64, p < 0.001). Similarly, the RLAI was significantly higher on the coasts than in the mountains (F = 4.48, p = 0.039). Climate drivers showed significant differences among the different forest types (e.g., effective annual precipitation, soil temperature and relative air humidity) and landscapes (e.g., soil moisture, effective annual precipitation and relative air humidity). Precipitation levels occurred from greatest to least in the following order: pure deciduous > mixed deciduous-evergreen > pure evergreen forests (F = 17.45, p < 0.001). Soil temperature was recorded in the forests in the following order from highest to lowest: pure evergreen > pure deciduous > mixed deciduous-evergreen forests (F = 3.84, p = 0.028), while relative air humidity was recorded in the forests in the following order from highest to lowest: mixed deciduous-evergreen > pure evergreen > pure deciduous forests (F = 51.52, p < 0.001). In addition, landscapes showed differences in soil moisture and precipitation, which were higher in the mountains (F = 135.07, p < 0.001) than on the coasts (F = 33.82, p < 0.001), and relative air humidity was higher in the coasts than on the mountains (F = 36.01, p < 0.001). The soil and forest floor characteristics also showed significant differences among the different forest types (e.g., slope, pH, resistance to compaction, bare soil, vascular plants cover) and between landscapes (e.g., bare soil, resistance to penetration, lichen cover). The slope (F = 4.95, p = 0.012) and bare soil (F = 10.64, p < 0.001) presented higher values in pure evergreen and mixed deciduous-evergreen forests than in pure deciduous forests. In contrast, pH (F = 30.14, p < 0.001) and resistance to compaction (F = 6.78, p = 0.002) were higher for pure deciduous forest > mixed deciduous-evergreen forests > pure evergreen forests. Vascular cover was recorded in the forests in the following order: pure deciduous > pure evergreen > mixed deciduous-evergreen forests (F = 31.67, p < 0.001). In addition, resistance to penetration (F = 100.36, p < 0.001), bare soil (F = 17.36, p < 0.001) and lichen cover (F = 5.50, p = 0.023) were higher on the coasts than in mountains, while pH (F = 8.32, p = 0.006) was higher in the mountains than on the coast. Regarding these analyses, there were significant interactions for basal area (F = 5.70, p = 0.006), effective annual precipitation (F = 33.82, p < 0.001), relative air humidity (F = 20.34, p < 0.001), pH (F = 3.87, p = 0.027), resistance to penetration (F = 3.20, p = 0.048), vascular cover (F = 4.88, p = 0.011) and lichen cover (F = 4.01, p = 0.024), (Table 1 and S1 Fig). On the other hand, basal area, dominant height and diameter at breast height did not significantly differ between deciduous and evergreen species in the mixed forests (S2 Table). These differences, except for diameter at breast height, were not detected for landscapes (coasts > mountains) (F = 4.44, p = 0.042). Interactions were also not significant (S2 Table). The interactions in the basal area that occurred for the different forest types had similar values, where pure deciduous forests had a significantly lower basal area than mixed deciduous-evergreen and pure evergreen forests in the mountains. The basal area in the coastal and mountain landscapes differed only in mixed deciduous-evergreen forests (S1 Fig). The effective annual precipitation was different among the different forest types, where annual precipitation in pure evergreen forests > mixed deciduous-evergreen > deciduous forests in the coasts; in contrast, annual precipitation in the mountains was higher, in deciduous forests than in mixed and evergreen forests. The relative air humidity values were similar in the pure deciduous forests in both landscapes. However, the mixed deciduous-evergreen and pure evergreen forests presented higher relative air humidity on the coasts than in the mountains (S1 Fig). In the pH analysis, a significant interaction was detected because all forest types had significant values on the coasts, but mixed deciduous-evergreen and pure evergreen forests had similar pH values in the mountains (S1 Fig). The resistance to penetration was similar in all forest types in the mountains, but on the coasts, resistance was significantly higher in pure deciduous than in mixed deciduous-evergreen and pure evergreen forests. However, the resistance to penetration was significantly higher on the coasts than in the mountains (S1 Fig). Vascular cover showed that pure deciduous forests had significantly different values between landscapes (mountains > coasts) (S1 Fig). Interactions in the lichen cover were significantly higher on the coasts than in the mountains in mixed deciduous-evergreen and pure evergreen forests (S1 Fig) but were not detected in pure deciduous forests.

**Table 1:** Generalized linear mixed models (GLMMs) to evaluate the effect of forest type (Np = pure deciduous forests, M = mixed deciduous-evergreen forests, Nb = pure evergreen forests) and landscapes (COA = coasts and, MOU = mountains) on. (i) forest structure: BA = basal area (m^2^ ha^-1^), DH = dominant height (m), DBH = diameter at breast height (cm), CC = canopy cover (%), and RLAI = relative leaf area index; (ii) climate: TR = transmitted total solar radiation (%), SM = soil moisture (%), PP = effective annual precipitation (mm yr^-1^), ST = soil temperature (°C), AT = air temperature (°C), and RH = relative air humidity (%); (iii) soil and forest floor characteristics: S = slope (%), pH = pH of the upper 10 cm of the soil, R = resistance to penetration (N cm^-2^), BS = bare soil cover (%), Ds = woody debris cover up to 5 cm diameter (%), and VC = vascular plant cover including ferns, monocots and dicots (%), L = lichen cover (%).

Factor		Forest structure					Climate						Soil and forest floor characteristics						
		BA	DH	DBH	CC	RLAI	TR	SM	PP	ST	AT	RH	S	pH	R	BS	Ds	VC	L
**Forest types**	Np	66.5 a	21.9 b	63.2 c	86.1	2.5 b	18.7	31.4	59.5 c	6.0 b	6.5	40.0 a	7.6 a	4.9 c	392.8 b	16.5 a	14.7	14.8 b	0.6
	M	77.1 b	17.6 a	47.9 b	86.2	2.5 b	18.5	34.9	53.4 b	4.5 a	6.5	63.3 c	12.7 b	4.3 b	288.3 a	38.8 b	17.7	6.5 a	0.9
	Nb	84.5 b	15.9 a	37.5 a	86.8	2.1 a	18.1	39.3	45.6 a	6.5 b	7.0	53.6 b	12.9 b	3.8 a	250.5 a	31.4 b	17.3	6.9 a	0.7
	F	7.12	12.31	33.35	0.67	10.64	0.39	1.47	17.45	3.84	0.27	51.52	4.95	30.14	6.78	10.64	1.14	31.67	0.59
	p	0.002	< 0.001	< 0.001	0.514	< 0.001	0.681	0.240	< 0.001	0.028	0.765	< 0.001	0.011	< 0.001	0.002	< 0.001	0.327	< 0.001	0.558
**Landscapes**	COA	71.4 a	18.7	51.7	86.2	2.5 b	18.8	13.4 a	58.3 b	5.9	7.0	57.3 b	10.1	4.1 a	474.2 b	36.6 b	17.3	8.5	1.0 b
	MOU	80.7 b	18.3	47.4	86.5	2.3 a	18.1	57.0 b	47.2 a	5.2	6.3	46.0 a	11.7	4.5 b	146.8 a	20.5 a	15.8	10.3	0.4 a
	F	5.80	0.17	2.88	2.56	4.48	0.91	135.07	33.82	1.30	1.05	36.01	0.61	8.32	100.36	17.36	0.68	3.53	5.50
	p	0.020	0.683	0.100	0.112	0.039	0.345	< 0.001	< 0.001	0.259	0.310	< 0.001	0.439	0.006	< 0.001	< 0.001	0.414	0.066	0.023
**Interactions**	F	5.70	1.80	0.78	3.10	1.63	2.89	2.84	66.64	0.35	0.27	20.34	1.05	3.87	3.20	0.23	0.78	4.88	4.01
	p	0.006	0.175	0.462	0.053	0.206	0.064	0.067	< 0.001	0.704	0.765	< 0.001	0.358	0.027	0.048	0.797	0.462	0.011	0.024

F, p = F test, probability. Different letters in each column show significant differences based on the LSD Fisher’s test at p < 0.05.

### Cover and richness of liverworts and mosses

Liverwort (F = 4.05, p = 0.023) and moss (F = 4.88, p = 0.011) cover showed significant differences among the different forest types, and the cover values were highest in pure evergreen, followed by mixed deciduous-evergreen and then pure deciduous forests. Moss cover showed significant differences between landscapes (mountains > coasts) (F = 14.79, p < 0.001) (Table 2).

**Table 2:** Generalized linear mixed models (GLMMs) to evaluate the effect of forest types (Np = pure deciduous forests, M = mixed deciduous-evergreen forests, Nb = pure evergreen forests) and landscapes (COA = coasts, MOU = mountains). Cover for liverworts and mosses (%).

		Cover	
Factor		Liverworts	Mosses
Forest types	Np	1.5 a	5.1 a
	M	3.7 ab	10.0 b
	Nb	5.0 b	10.7 b
	F	4.05	4.88
	p	0.023	0.011
Landscapes	COA	2.9	5.5 a
	MOU	3.9	11.7 b
	F	1.10	14.79
	p	0.298	< 0.001
Interaction	F	2.39	3.10
	p	0.102	0.053

F, p = F test, probability. Different letters in each column show significant differences based on the LSD Fisher’s test at p < 0.05.

**Fig 2:**
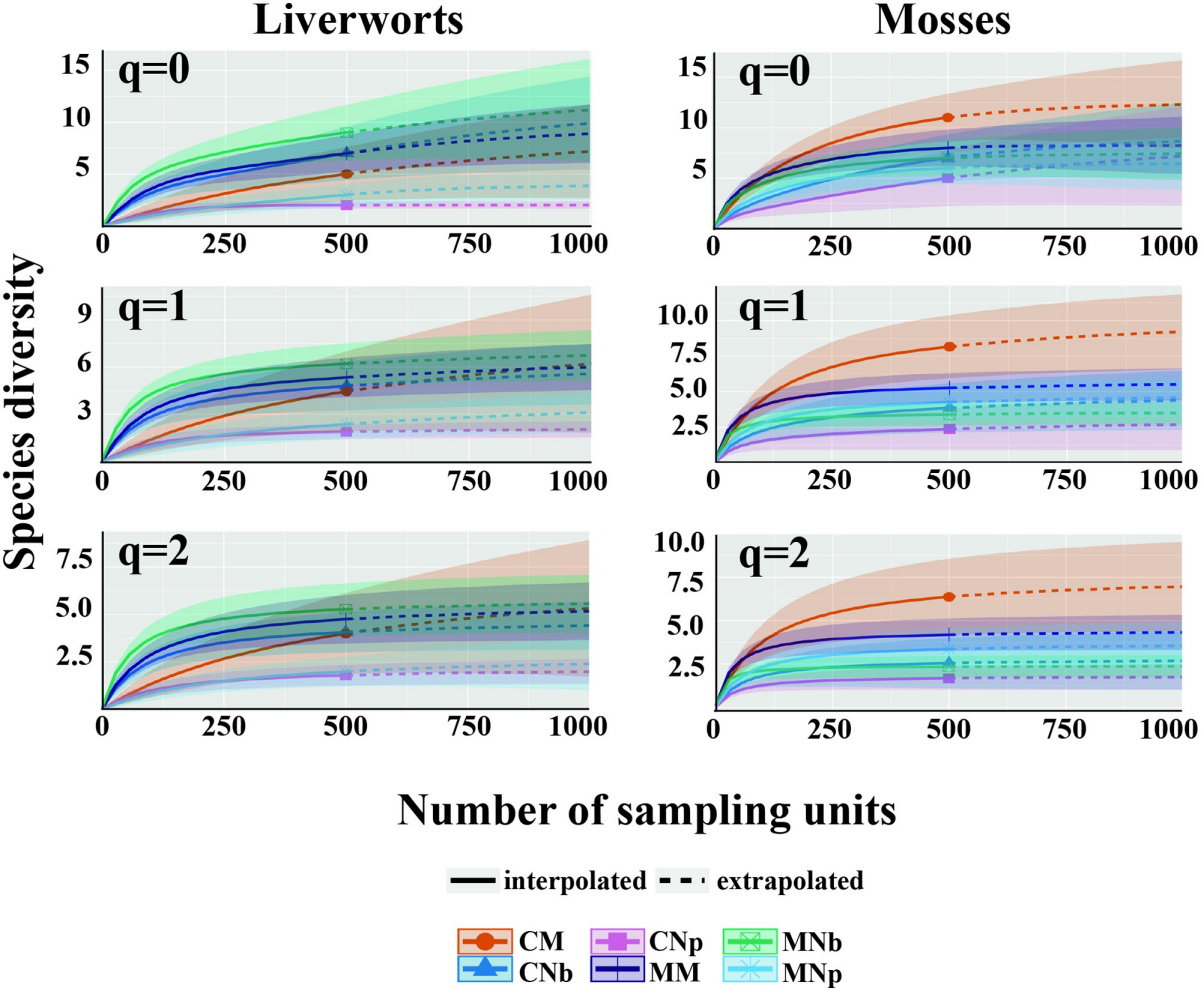
Comparisons of sample-size-based rarefaction (solid segment) and extrapolations (dotted line segments) of liverworts and mosses for different Hill numbers (q = 0, q = 1, q = 2) for the different forest types and landscapes. The 95% confidence intervals were obtained by a bootstrap method based on 100 replications (shaded areas). Pure deciduous *N*. *pumilio* forests in the coasts (CNp) and mountains (MNp), pure evergreen *N*. *betuloides* forests in the coasts (CNb) and mountains (MNb), mixed deciduous-evergreen forests in the coasts (CM) and mountains (CM). The solid dots and the other symbols represent the reference samples.

We constructed a sample size-based rarefaction and extrapolation sampling curve (Fig 2) that showed the trend in Hill numbers when the number of sampling units increased. The species richness curves showed minor differences (overlapping confidence intervals) among the forest types in each landscape. All confidence intervals overlapped, implying that diversity of any order q = 0, 1, and 2 was not significantly different among the forest types and landscapes for any sample size up to 1000 sampling units. In liverworts, the level of richness was greatest in the pure evergreen forests in the mountains, followed by mixed deciduous-evergreen forests in the mountains and then pure evergreen forests on the coasts, exceeding the liverwort richness that occurred in the following order: mixed deciduous-evergreen forests on the coasts > pure deciduous forests in the mountains > pure deciduous forests on the coasts. For mosses, the richness values in the forests occurred in the following order: mixed deciduous-evergreen forests on the coasts > mixed deciduous-evergreen forests in the mountains > pure deciduous forests in the mountains > pure evergreen forests in the mountains > pure evergreen forests on the coasts > pure deciduous forests on the coasts. The effective number of species calculated by the Hill number q = 0 approached the asymptote after extrapolation. All three Hill numbers increased consistently among the forest types and landscapes, except for liverworts in pure deciduous forests on the coasts, which did not increase after extrapolation (they reached the asymptote) (Fig 2 and S3 Table). However, there was a decrease in the diversity values (q = 0 > q = 1 > q = 2), mainly for liverworts. Shannon’s and Simpson’s diversity indexes were not asymptotic, with increased values even when they were extrapolated. The trends in the increases were higher for liverworts and mosses mainly in mixed deciduous-evergreen forests on the coasts than in the mountains. We could have obtained a greater number of species than those collected if the model sampling effort was increased (e.g., if the number of transects increased, then the richness would also increase). More details for estimated diversity and estimated sample coverage were informed in S3 Table.

### Similarity index in pure and mixed forests in different landscapes

**Fig 3:**
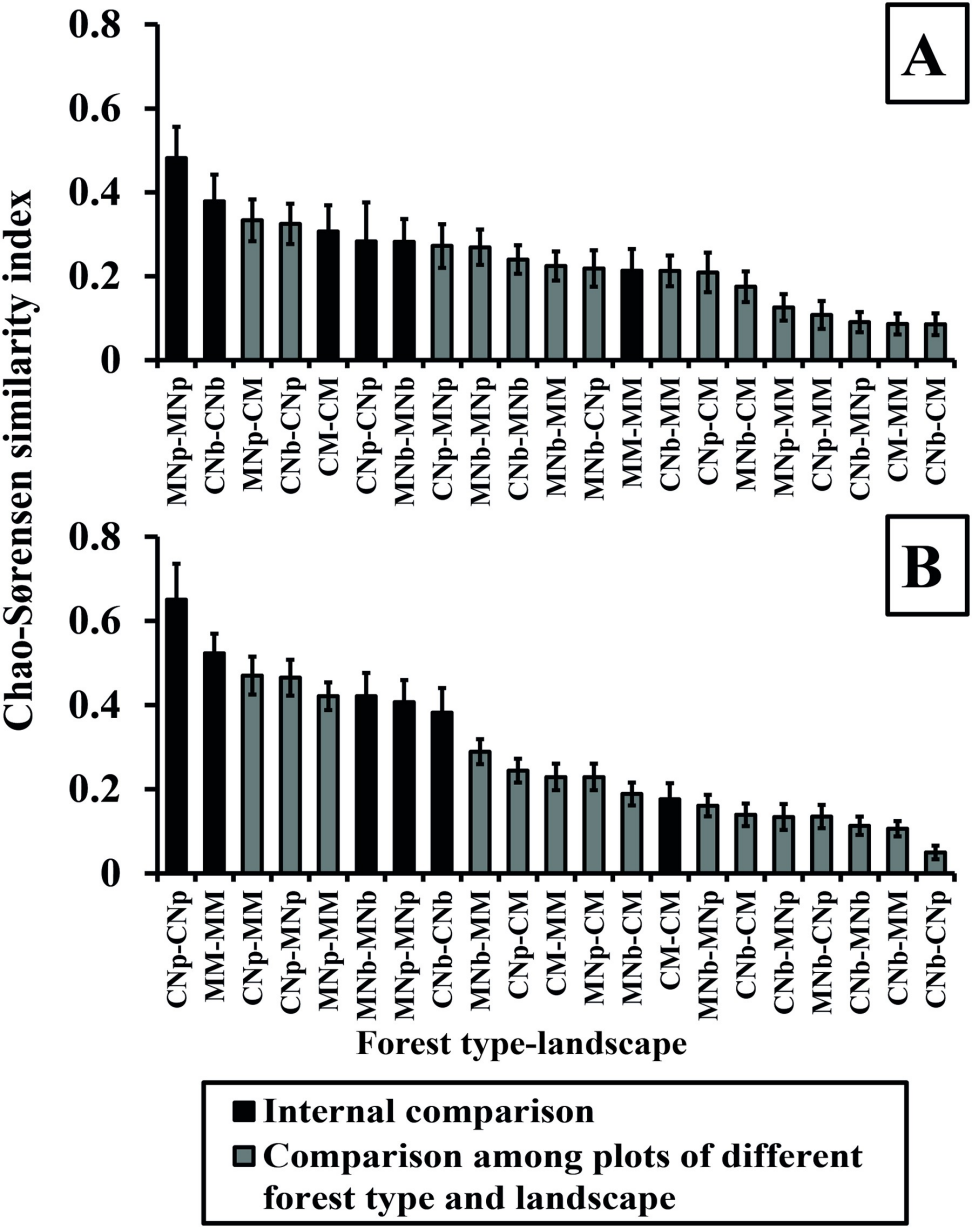
The incidence based on the Chao-Sørensen similarity index for liverworts (A) and mosses (B) at each forest type and landscapes. Pure deciduous *N*. *pumilio* forests in the coasts (CNp) and mountains (MNp), pure evergreen *N*. *betuloides* forests in the coasts (CNb) and mountains (MNb), mixed deciduous-evergreen forests in the coasts (CM) and mountains (MM). Bars in black correspond to internal comparisons for each forest type and landscapes, while bars in gray correspond to comparisons among plots of different forest types and landscapes. Error bars correspond to standard errors of the mean.

Fig 3 shows the incidence‐based Chao-Sørensen similarity index between comparisons of forest types and landscapes, including self-comparisons, both for liverworts and mosses. For liverworts (Fig 3A), the internal similarity varied between 0.21 and 0.48, with comparisons between pure deciduous forests in the mountains (MNp vs MNp), pure evergreen forests on the coasts (CNb vs CNb), and mixed deciduous-evergreen forests on the coasts (CM vs CM) with the major index values and the pure deciduous forests on the coasts (CNp vs CNp), pure evergreen forests in the mountains (MNb vs MNb), and mixed deciduous-evergreen forest in the mountains (MM vs MM) with the minor index values. For the comparison between the plots of different forest types and landscapes, the comparisons of pure deciduous forests in the mountains and mixed deciduous-evergreen forests on the coasts (MNp vs CM), pure evergreen and pure deciduous forests on the coasts (CNb vs CNp) and pure deciduous forests on the coasts and pure deciduous forests in the mountains (CNp vs MNp) had the greatest similarity index values, while those of the pure evergreen forests on the coasts and pure deciduous forests in the mountains (CNb vs MNp), mixed deciduous-evergreen forests on the coasts and mixed deciduous-evergreen in the mountains (CM vs MM), and pure evergreen forests and mixed deciduous-evergreen forests on the coasts (CNb vs CM) had the least similarity index values.

For mosses (Fig 3B), the internal similarity varied between 0.18 and 0.65, with the comparisons between plots of pure deciduous forests on the coasts (CNp vs CNp), mixed deciduous-evergreen forests in the mountains (MM vs MM), and pure evergreen forests in the mountains (MNb vs MNb) with the major index values and the pure deciduous forests in the mountains (MNp vs MNp), pure evergreen forests on the coasts (CNb vs CNb), and mixed deciduous-evergreen forest on the coasts (CM vs CM) with the minor index values. For the comparisons between the plots of different forest types and landscapes, the comparisons of pure deciduous forests on the coasts and mixed deciduous-evergreen in the mountains (CNp vs MM), pure deciduous forests on the coasts and pure deciduous forests in the mountains (CNp vs MNp), and pure deciduous forests and mixed deciduous-evergreen forests in the mountains (MNp vs MM) presented the greatest similarity index values, while those of pure evergreen forests on the coasts and pure evergreen forests in the mountains (CNb vs MNb), pure evergreen forests on the coasts and mixed deciduous-evergreen forests in the mountains (CNb vs MM) and pure evergreen forests and pure deciduous forests on the coasts (CNb vs CNp) presented the least similarity index values.

We did not find statistically significant nestedness among the whole study treatments, neither for forest type in each landscape nor for landscapes for each forest type for both the moss and liverwort comparisons (Table 3). The NODF values were lower than 28.0 for the liverworts and 38.8 for the mosses. Similarly, the NODF value for forest types × landscapes was consistently higher for the whole study, within each landscape type, and for pure deciduous forest and mixed forests, while the NODF value for species was higher in pure evergreen forests between coasts and mountains, both for liverworts and mosses.

**Table 3:** Nestedness analyses for liverwort and moss species.

	Liverworts					Mosses				
*Whole sites comparison (forest types × landscapes)*										
METRIC	Fill (%)	INDEX	Z-SCORE	RN	NESTED	Fill (%)	INDEX	Z-SCORE	RN	NESTED
**NODF**	14.1	20.854	-0.869	-0.036	No (p > 0.05)	14.0	27.364	0.726	0.023	No (p > 0.05)
**NODF_species**		13.069	-1.099	-0.122	No (p > 0.05)		19.727	0.117	0.011	No (p > 0.05)
**NODF_sites**		21.547	-0.731	-0.031	No (p > 0.05)		27.884	0.733	0.023	No (p > 0.05)
*Coasts comparison among forest types*										
**NODF**	16.7	20.796	-0.598	-0.042	No (p > 0.05)	14.8	19.851	0.611	0.043	No (p > 0.05)
**NODF_species**		15.278	0.554	0.110	No (p > 0.05)		18.483	0.868	0.114	No (p > 0.05)
**NODF_sites**		21.842	-0.826	-0.059	No (p > 0.05)		20.179	0.362	0.028	No (p>0.05)
*Mountains comparison among forest types*										
**NODF**	18.9	28.042	0.616	0.040	No (p > 0.05)	25.0	38.503	-0.296	-0.009	No (p > 0.05)
**NODF_species**		17.196	-1.815	-0.221	No (p > 0.05)		35.463	0.489	0.033	No (p > 0.05)
**NODF_sites**		30.897	1.351	0.094	No (p > 0.05)		38.755	-0.383	-0.012	No (p > 0.05)
*Pure deciduous forest comparison between landscapes*										
**NODF**	31.3	27.451	0.904	0.118	No (p > 0.05)	20.6	38.821	-0.243	-0.007	No (p > 0.05)
**NODF_species**		22.222	0.126	0.046	No (p > 0.05)		22.024	-0.453	-0.056	No (p > 0.05)
**NODF_sites**		28.571	0.762	0.131	No (p > 0.05)		42.279	-0.044	-0.001	No (p > 0.05)
*Mixed forests comparison between landscapes*										
**NODF**	17.1	18.444	0.262	0.025	No (p > 0.05)	18.2	30.512	0.016	0.001	No (p > 0.05)
**NODF_species**		17.963	-0.271	-0.042	No (p > 0.05)		24.254	-0.681	-0.055	No (p > 0.05)
**NODF_sites**		18.681	0.52	0.060	No (p > 0.05)		33.509	0.337	0.022	No (p > 0.05)
*Pure evergreen forest comparison between landscapes*										
**NODF**	20.9	16.909	-0.114	-0.020	No (p > 0.05)	20.0	28.571	0.937	0.061	No (p > 0.05)
**NODF_species**		18.287	-0.203	-0.021	No (p > 0.05)		28.995	1.550	0.176	No (p > 0.05)
**NODF_sites**		16.544	-0.089	-0.019	No (p > 0.05)		28.46	0.475	0.034	No (p > 0.05)

NODF = nestedness measurement based on overlapping and decreasing fills; INDEX = nestedness index; Z SCORE = statistic for the null model; RN = relative nestedness; and NESTED = evaluation of nestedness and probability level. Sites = forest types and landscapes.

### Indicator species in each forest type and landscape

The indicator species showed that among the moss species, *Dicranoloma robustum* and *Ditrichum cylindricarpum* were more frequent in evergreen forests, while *Acrocladium auriculatum* was more frequent in deciduous forests and *Campylopus clavatus* in mixed deciduous-evergreen forests (Table 4). Regarding landscapes, the liverwort *Leptoscyphus huidobroanus* and the moss *Dicranoloma robustum* were indicator species in coastal environments, while the mosses *Campylopus clavatus* and *Ditrichum cylindricarpum* were indicator species in the mountains (Table 4).

**Table 4:** Indicator species analyses for bryophyte composition in each forest type and each landscape.

Forest type			Landscape	
Pure deciduous forests	Mixed deciduous-evergreen forests	Pure evergreen forests	Coasts	Mountains
*Acrocladium auriculatum*	*Campylopus clavatus*	*Dicranoloma robustum*	*Leptoscyphus huidobroanus*	*Campylopus clavatus*
(38.6)*	(25.0)*	(30.0)*	(34.6)**	(25.8)**
		*Ditrichum cylindricarpum*	*Dicranoloma robustum*	*Ditrichum cylindricarpum*
		(28.9)*	(30.4)*	(30.4)*

The indicator values are reported between brackets.

* = significance at p < 0.05,

** = indicated significance at p < 0.01.

### Drivers that influence liverworts and mosses in each forest type and each landscape

**Fig 4:**
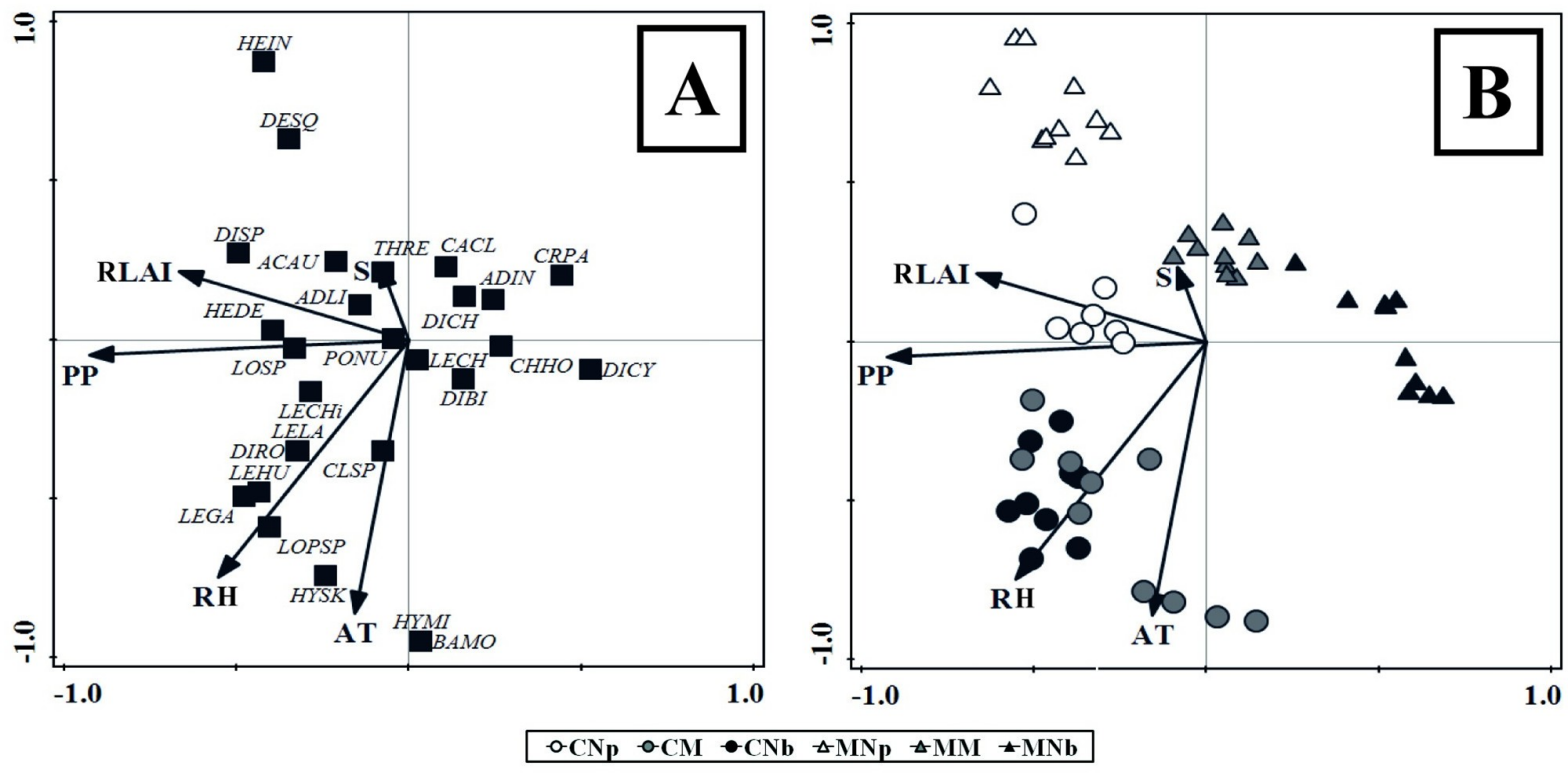
Canonical correspondence analysis (CCAs) based on species abundance to assess the influence of the analyzed environmental drivers. RLAI = relative leaf area index, S = Slope (%), PP = effective annual precipitation (mm yr^-1^), RH = relative air humidity (%), and AT = air temperature (°C) influence on (A) bryophyte species distribution (see S1 Table for species code), and (B) forest types and landscapes: pure deciduous *N*. *pumilio* forests in the coasts (CNp) and mountains (MNp), pure evergreen *N*. *betuloides* forests in the coasts (CNb) and mountains (MNb), mixed forests in the coasts (CM) and mountains (MM).

The CCAs explained 67% of the variance for the species-environment, with a total inertia of 7.3 and eigenvalues of 0.485 for axis 1 and 0.366 for axis 2 (Fig 4). The environmental drivers employed for the CCAs were previously selected for statistical significance according to the Pearson correlation coefficient (Table 5 and S4 Table). Axis 1 was influenced by the effective annual precipitation and RLAI, while axis 2 was influenced by the relative air humidity, air temperature and slope. When species were analyzed alone (Fig 4A), both axes showed a close correlation and a clear association between mosses and liverworts according to the studied microclimatic drivers. Moreover, the CCAs separated the sampling plots into two main groups, defined by landscape (coasts and mountains), showing few differences among the different forest types (Fig 4B). The environmental drivers were most related to the forests on the coasts (e.g., RLAI, effective annual precipitation, relative air humidity, air temperature), while slope was influenced by the forests in the mountains.

**Table 5:** Significant drivers obtained in the canonical correspondence analysis, including explained variation, contribution, pseudo-F test, and associated probability p < 0.05.

Drivers	Variation explained %	Contribution %	pseudo-F	p
PP	6.1	32.2	3.6	0.002
RH	4.5	23.7	2.7	0.002
RLAI	2.9	15.1	1.8	0.012
AT	2.9	15.2	1.8	0.022
S	2.6	13.9	1.7	0.020

PP = effective annual precipitation (mm yr^-1^), RH = relative air humidity (%), RLAI = relative leaf area index, AT = air temperature (°C), and S = slope (%).

The liverworts were found over all substrates, and they did not present significant differences in their associations with substrate or forest type and landscape (chi-square = 22.9, df = 20, p = 0.289). In contrast, mosses presented significant differences in their associations (chi-square = 77.08, df = 25, p < 0.001). In comparison to other species, the moss *Acrocladium auriculatum* and the liverworts *Adelanthus lindbergianus* and *Lepidozia chordulifera* occurred on a higher variety of substrates, as they were not found only on stones (S5 Table and S2 Fig). The most common moss species *Acrocladium auriculatum* was significantly associated (chi-square = 28.9, df = 12, p = 0.004) with litter, decaying wood, bare soil, and epiphytes, considering the different forest types and landscapes (except for pure evergreen forests on the coasts, where this species was not found). Another moss, *Dicranoloma robustum*, was significantly associated (chi-square = 18.2, df = 5, p = 0.003) with two substrates, litter and decaying wood, and with all forest types in different landscapes. Of the most common liverworts, *Leptocyphus huidobroanus* was found only in the forests growing on the coasts and was statistically associated (chi-square = 16.1, df = 6, p = 0.013) with litter, decaying wood, bare soil and epiphytes.

## Discussion

### Drivers that most influence bryophytes

Our results indicated that bryophytes are mainly influenced by a combination of microclimate conditions, which may vary according to forest tree composition. Therefore, hypothesis (i) was partially accepted. Our results are in agreement with previous findings of studies conducted on other continents (e.g., North America, Europe and Asia), where bryophyte cover, richness and diversity varies among forest types or stand conditions (e.g., post harvesting or natural disturbances) due to specific biotic and abiotic drivers [1, 6, 8, 55, 56].

The CCAs separated the sampling plots into two main groups, mainly defined by their landscapes. Air temperature and relative air humidity seemed to be highly important to bryophyte composition in the studied gradients in each forest type and landscape. Precipitation was also crucial due to its influence on water storage in soils and environmental moisture. However, the water storage capacity differed between soils of evergreen and deciduous *Nothofagus* forests [24], which could generate differences in the soil moisture and therefore differences in the bryophyte cover, as was suggested by Raabe et al. [7]. In addition, air temperature and relative air humidity are considered key drivers in structuring bryophyte communities in different forest types [7, 55]. This concept is very important, because previous studies have shown that the availability of atmospheric water is more important than the level of soil moisture for bryophytes since bryophytes absorb water throughout their entire plant structure [57]. On the other hand, in comparison to the mountain forests, the coastal forest environments had higher temperatures and higher air humidity, while mountains had greater soil moisture maintaining favorable microclimatic conditions for bryophyte development. Therefore, our results reaffirm that a high level of air humidity surrounding a forest understory is important for bryophytes as are, shaded conditions [8]. Our study showed that the relative air humidity was higher on the coasts (influenced by the proximity to the sea) than in mountains, and this variable most influenced the cover, richness and diversity of bryophytes (mainly in pure evergreen forests and mixed deciduous-evergreen forests), while soil moisture better explained the specific composition of forests in the mountains.

Changes in forest structure and RLAI in deciduous forests throughout the year (e.g., complete foliage development in summer and loss of 50% foliage in autumn-winter) resulted in a higher variation in solar radiation reaching the ground, wind exposure, and air and soil moisture than that in the other forests [5]. Within mixed and evergreen forests, where more than half of the canopy remained year-round, these microclimatic drivers were more stable than those in the other forest types. Tinya et al. [58] found that solar radiation correlates with bryophyte richness on the forest floor, although direct exposure to radiation can also cause desiccation. In coniferous forests, solar radiation benefits bryophyte richness due to the availability of additional radiation at the forest floor because of a less- closed canopy and a less litter drop compared to that in deciduous forests [58]. However, in our study, solar radiation was not considered the major driver of bryophyte composition. These differences can occur in specific places (e.g., gaps) that were not specifically considered in this study, but it is an interesting subject to consider in future studies.

Marialigeti et al. [12] showed that the litter of deciduous species changes the properties of soils, which can inhibit bryophyte development. We observed that bare soil and litter were the substrates with the largest number of liverworts and mosses in all forest types and landscapes. In the study area, Toro Manríquez et al. [24] calculated that the litterfall for deciduous forests exceeded 26.4% for mixed forests and 51.0% for evergreen forests. Therefore, this environmental driver could be essential for bryophyte composition. According to Müller et al. [55], different substrates in beech forests of central Europe favor vascular plants but are less suitable for bryophyte occurrence. On the other hand, bryophyte cover is negatively correlated with vascular plants species richness [59]. This scenario could explain why in our study, there was less bryophyte cover in the deciduous forests, which have greater vascular plant and lichen cover, than in the mixed and evergreen forests.

### Bryophyte cover and richness in each forest type and landscape

Studies on bryophytes in pure and mixed forests in southern Patagonia are scarce, and the knowledge available on mosses or liverworts mainly references other natural environments in Patagonia [60, 61]. A key result of our study, consistent with our hypothesis (ii) indicates that forest types and landscapes determined bryophyte cover and richness. Liverwort and moss covers showed a clear gradient from evergreen forests to mixed forests to deciduous forests. Thus, the mixed forests represented an intermediate condition between both pure forest types. Moreover, there was greater moss cover in the mountain forests than in the coastal forests, which could be related to the high soil moisture in the mountain forests, as was also described in other studies [7]. Based on the Hill number rarefaction-extrapolation curve, we reached a greater and more stable value of species richness in pure deciduous forests than in the other forest types. The increasing trend was higher for liverworts and mosses in the mixed deciduous-evergreen forests growing on the coasts than in the other forest types. Moreover, if the sampling effort increased (e.g., the number of transects), then richness also increased. This curve illustrates how much sampling effort is needed to achieve a predetermined level of sample completeness [45]. More studies in areas under other topographic conditions (e.g., steep slopes and cliffs) could provide more insight into rare species inhabiting hard environmental conditions. Considering a global scale, the total richness of liverworts (12 species) and mosses (15 species) recorded in our study was very low, e.g., mixed forests in North Europe have over 33 bryophyte species [62, 63], while old-growth Mediterranean forests sustain more than 36 bryophyte species [64]. This information motivates continued studies in search of endemic species, habitat-specific or species to sensitive to habitat disturbances in Tierra del Fuego. Of the forest types, the pure deciduous forest on the coast had the lowest richness. Notably, the deciduous and mixed forests presented similar bryophyte richness regardless of the landscape, whereas the bryophyte richness in the evergreen forests was higher in specific landscapes (e.g., mountains rather than coasts). In other studies, these changes were also associated with environmental gradients, showing high habitat specificity for bryophyte species under different forest conditions [1, 9, 10].

## Bryophyte similarities in forest types and landscapes

Previous studies have reported that diversity increases with elevation [65], considering natural forest distributions. We related this statement to hypothesis (iii), because the most diversity occurred in the mountain forests. However, with the Chao-Sørensen diversity analysis [47], it was possible to analyze self-comparisons among the plots with the same forest type and landscape, observing the dissimilarities among them, e.g., for liverworts, the similarity among the plots of the pure forests in the mountains and the dissimilarity among the plots of the forests on the coast. In contrast, the similarity of the plots in the pure evergreen forests on the coasts was compared with that in the pure evergreen forests in the mountains for liverworts, but the opposite trend was observed for mosses. These results indicated that both forest types and landscapes induced high habitat specificity for bryophyte species. However, habitat specificity for bryophytes in the evergreen forests was low, resulting in a rejection of hypothesis (iii). While pure deciduous forests were similar in terms of species richness between the coast and mountains, they presented different species assemblages (e.g., differences associated with drivers such as soil moisture and air humidity that differed between the coasts and mountains). Hence, these differences might also affect dispersal abilities of liverworts and mosses, as well as species provenance. Specifically, bryophyte species may be distributed even in a homogeneous environment [1]. The low proportion of shared species among the plots and forest types within each landscape showed that each forest type and landscape hosted unique biodiversity.

Nestedness analysis is usually used to identify gradients that can influence species, including bryophytes [66], but in this study, we found no-nestedness for each forest type and landscape. We did not determine whether one bryophyte community was a subset of another community through this analysis, and therefore, we cannot conclude whether bryophytes in one mixed forest are a subset of bryophytes from the pure forests. This result can be explained through the analysis of diversity that showed that the set of bryophytes varied even among plots of the same forest type and landscape. However, the understanding of nestedness patterns deserves more analysis to test whether high segregation exists, as well as whether species turnover or other patterns [50] could explain the liverwort and moss species distributions in the coastal and mountainous natural environments of Tierra del Fuego. These analyses rarely incorporate the functional traits of species or specific environmental characteristics of sampled sites, even though the outcomes of species interactions often depend on trait expression and site quality [67]. Thus, it is necessary to explore more specific stand conditions (e.g., steep slopes and higher altitudes) and compare them with those in other adjacent areas where these forest types occur (e.g., biogeographic patterns of species occurrence) to better understand species occurrence and/or associations [68].

As mentioned before, the role of microclimate and the diversity or frequency of different substrates could contribute to the occurrence of specific indicator species in different forest types [1, 12, 20]. In our study, we reported that mixed forests have more exclusive species than pure deciduous or evergreen forests, and therefore we must reject hypothesis (iv). Although the exclusivity of species was low in the evergreen forest, this forest type showed that the highest species richness can be explained by decaying wood coverage, which was essential for bryophyte occurrence. In contrast, the deciduous forests the more essential substrate was litter. This result is important to consider when implementing conservation strategies, because the heterogeneity of old-growth forests contributes to the substrate diversity where bryophytes can occur [12, 56]. In future studies (e.g., on conservation management), the contribution of substrate diversity in old-growth forests must be explored and linked to bryophyte occurrence and frequency, which are important to consider in the development of new conservation strategies. This concept highlights the importance of conservation actions at a local scale (e.g., specific proposals for each forest type) focused on the bryophyte community avoiding large-scale canopy disturbances (e.g., opening or expanding new routes) to prevent potential microclimate alterations (e.g., air temperature, air humidity, precipitation or soil conditions in the face future disturbances), which can decrease the number of certain sensitive species or cause local extinctions.

## Conclusions

Here, the influence of forest canopy-layer composition (pure or mixed species) on the structure (cover) and composition (richness and diversity) of bryophytes was explored to understand how environmental variability can affect understory vegetation in coastal and mountainous environments. The studied treatments (e.g., forest types and landscapes) played a crucial role in the bryophyte (liverworts and mosses) cover, richness, and composition. The studied environmental drivers were mainly explained by the microclimate, with a higher effective annual precipitation and relative air humidity in the coastal forests than in the mountainous forests and higher soil moisture in the mountainous forests. However, most of the studied variables did not explain bryophyte diversity, which in turn was mostly linked to soil and substrate (e.g., woody debris). In addition, in comparison to the coast, the mountains have greater bryophyte diversity and cover for all forest types. Our results suggest that pure evergreen and mixed deciduous-evergreen forests in the mountains rather than on the coast supported higher moss cover, while the deciduous forests in both landscapes were similar. A greater similarity of liverwort species was found in mixed deciduous-evergreen forests in mountains and pure evergreen forests on the coasts than in the other forests. These outputs also highlight the need to explore differences at larger altitudinal ranges to implement sustainable management and conservation planning for bryophytes in the southernmost forests.

## Supporting information

S1 Fig Interactions corresponding to Table 1. BA = basal area (m^2^ ha^-1^), PP = effective annual precipitation (mm yr^-1^), RH = relative air humidity (%), pH = pH of the upper 10 cm of the soil, R = resistance to penetration (N cm^-2^), VC = vascular plant cover including ferns, monocots and dicots (%), L = lichen cover (%). Different letters showed significant differences by LSD Fisher test (p < 0.05). Lower cases were used for comparisons among forest types (Np = pure deciduous forests, M = mixed deciduous-evergreen forests, Nb = pure evergreen forests), and capital letters were used for comparisons between landscapes (COA = coasts, MOU = mountains).
(TIF)

S2 Fig Cover (%) of bryophyte species sampled for each substrate. (A) Liverworts and (B) mosses, analysing LT = litter cover (%), DW = decaying wood (%), BS = bare soil (%), EP = epiphytic on branches and bark in the forest floor (%), St = stones. Species codes are presented in S1 Table.
(TIF)

S1 Table Bryophyte species observed in each forest type. (Np = pure deciduous forests, M = mixed deciduous-evergreen forests, Nb = pure evergreen forests) and landscapes (COA = coasts, MOU = mountains), showing: (i) species code, (ii) TAX = taxonomic group (Li = liverworts, Ms = mosses), (iii) GDP = global distribution patterns (D = disjunct with South America, South Africa and Europe; E = endemic; PAN = pantropical-type *Podocarpus*; A = austral-Antarctic; COS = cosmopolitan; B = bipolar), and (iv) substrates (LT = litter; DW = decaying wood; BS = bare soil; St = stones; EP = epiphytic on branches and bark in the forest floor). OF = occurrence frequency in each forest type and landscapes (%), and ẋ = mean frequency of occurrence in the entire study (%).
(DOCX)

S2 Table Generalized linear mixed models (GLMMs) to evaluate the effect of tree species contribution in the canopy composition in mixed deciduous (*N*. *pumilio*) and evergreen (*N*. *betuloides*) forests at different landscapes (COA = coasts, MOU = mountains) over the following forest structure variables. BA = basal area (m^2^ ha^-1^), DH = dominant height (m), DBH = diameter at breast height (cm).
(DOCX)

S3 Table Rarefaction and extrapolation data through the estimated diversity by Hill number (q = 0, 1, 2) of order q for a sample size 500 and 1000. The estimated sample coverage for a sample size 500 and 1000 (sample size Observed = 500 and Expected by extrapolation = 1000) were presented between brackets. Pure deciduous *N*. *pumilio* forests in the coasts (CNp) and mountains (MNp), pure evergreen *N*. *betuloides* forests in the coasts (CNb) and mountains (MNb), mixed deciduous-evergreen forests in the coasts (CM) and mountains (CM).
(DOCX)

S4 Table Pearson correlation coefficients obtained between the tested variables of the canonical correspondence analysis. BA = basal area (m^2^ ha^-1^), DH = dominant height (m), DBH = diameter at breast height (cm), CC = canopy cover (%), RLAI = relative leaf area index, TR = transmitted total solar radiation (%), SM = soil moisture (%), PP = effective annual precipitation (mm yr^-1^), ST = soil temperature (°C), AT = air temperature (°C), RH = relative air humidity (%), S = slope (%), pH, R = resistance to penetration (N cm^-2^), BS = bare soil cover (%), Ds = debris cover (%), VC = vascular plant cover including ferns, monocots and dicots (%), and L = lichen cover (%). Correlation coefficients varied between -1 to +1. * = showed correlations values over 0.5 and under -0.5, with p-values < 0.05.
(DOCX)

S5 Table Crosstabs of frequency and chi-square test of liverworts and mosses for each substrate. LT = litter, DW = decaying wood, BS = bare soil, St = stones, EP = epiphyte of branches and bark in the forest floor. Pure deciduous *N*. *pumilio* forests in the coasts (CNp) and mountains (MNp), pure evergreen *N*. *betuloides* forests in the coasts (CNb) and mountains (MNb), mixed deciduous-evergreen forests in the coasts (CM) and mountains (CM).
(DOCX)
