# Supplementary figures and images for: Forest canopy-cover composition and landscape influence on bryophyte communities in Nothofagus forests of southern Patagonia
Source: PLoS One. 2020 Nov 24;15(11):e0232922. doi: 10.1371/journal.pone.0232922 (PMC7685467; doi:10.1371/journal.pone.0232922)

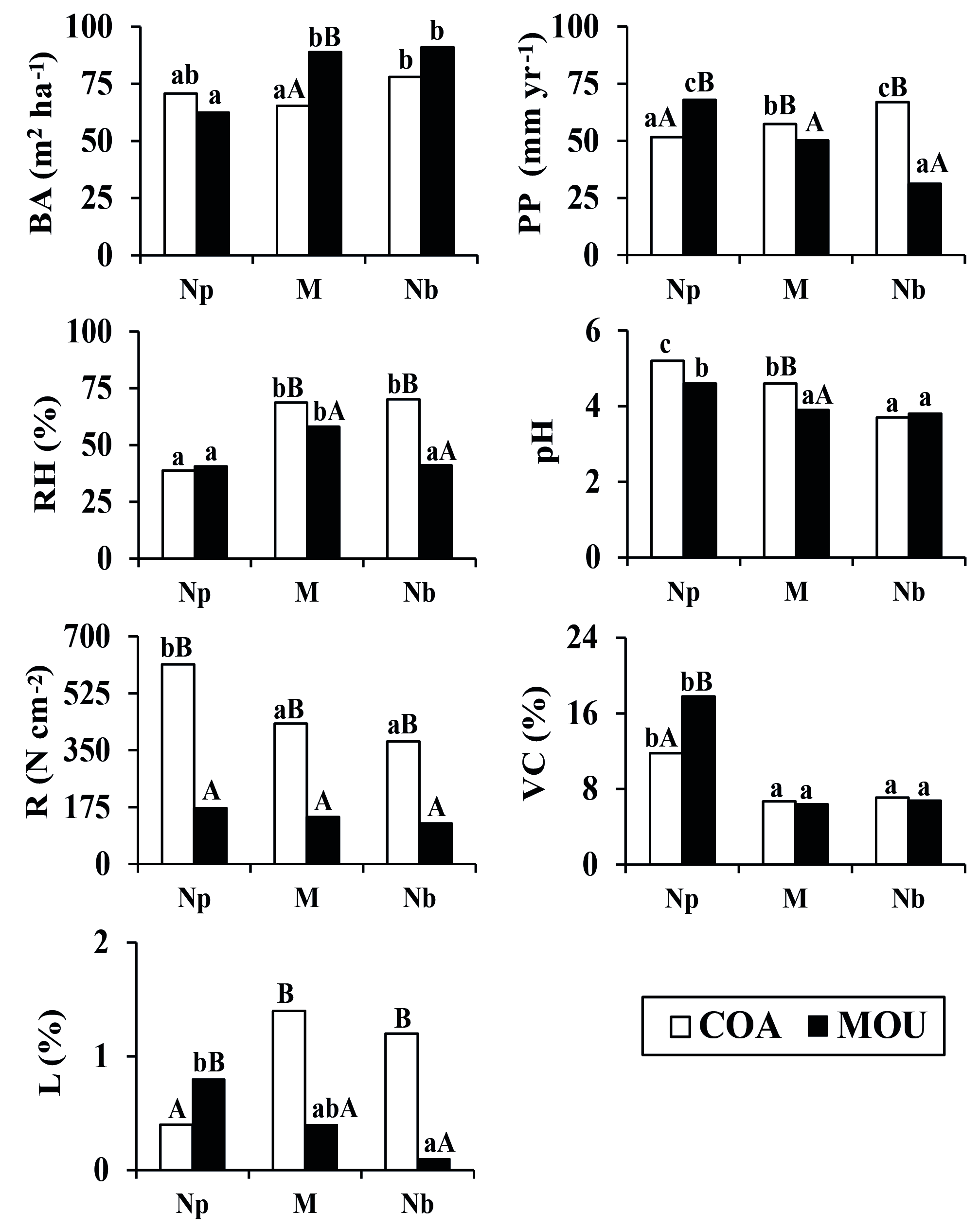

Supplement: S1 Fig — BA = basal area (m2 ha-1), PP = effective annual precipitation (mm yr-1), RH = relative air humidity (%), pH = pH of the upper 10 cm of the soil, R = resistance to penetration (N cm-2), VC = vascular plant cover including ferns, monocots and dicots (%), L = lichen cover (%). Different letters showed significant differences by LSD Fisher test (p < 0.05). Lower cases were used for comparisons among forest types (Np = pure deciduous forests, M = mixed deciduous-evergreen forests, Nb = pure evergreen forests), and capital letters were used for comparisons between landscapes (COA = coasts, MOU = mountains). (TIF) [file pone.0232922.s001.tif]

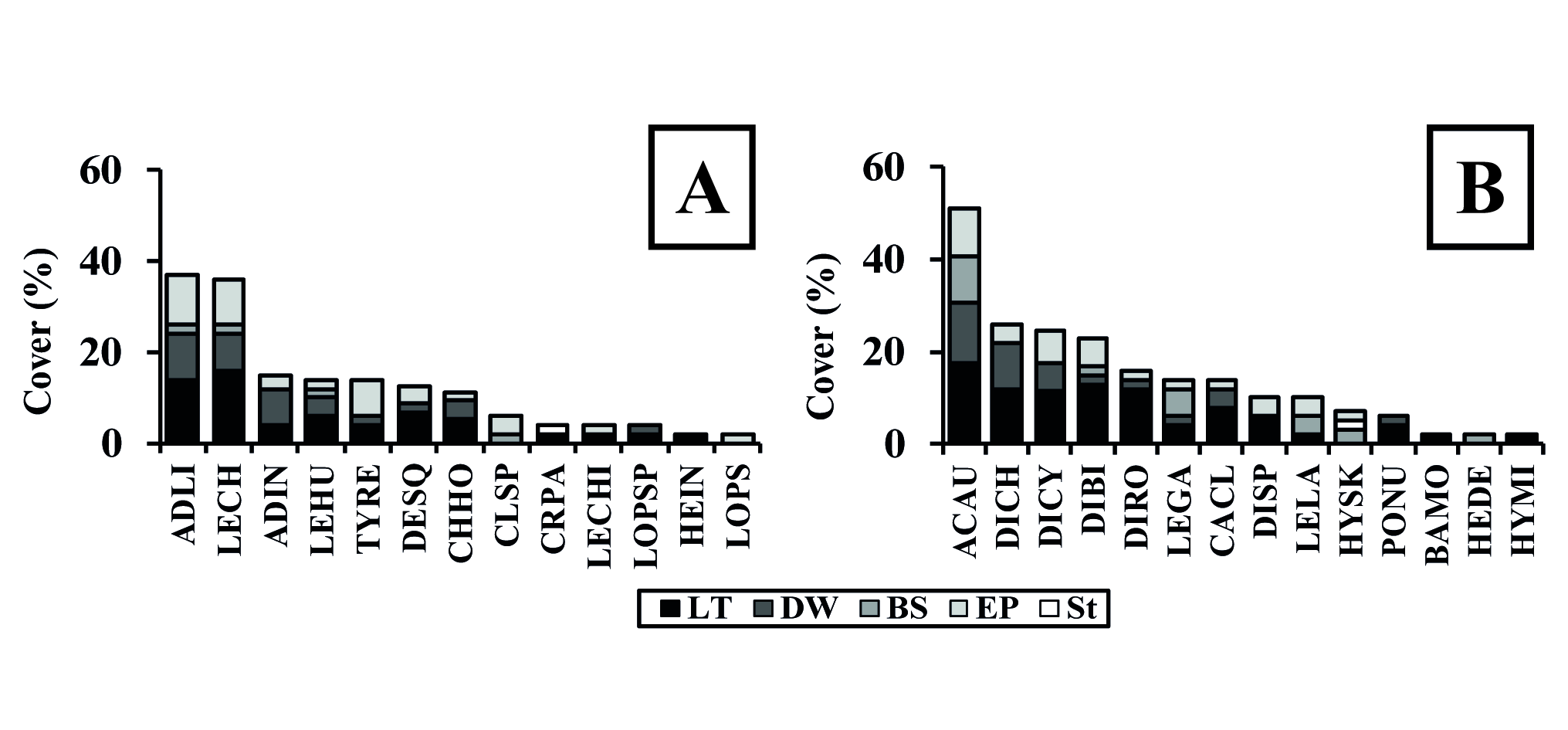

Supplement: S2 Fig — (A) Liverworts and (B) mosses, analysing LT = litter cover (%), DW = decaying wood (%), BS = bare soil (%), EP = epiphytic on branches and bark in the forest floor (%), St = stones. Species codes are presented in S1 Table. (TIF) [file pone.0232922.s002.tif]
