# Supplementary material for: Forest canopy-cover composition and landscape influence on bryophyte communities in Nothofagus forests of southern Patagonia
Source: PLoS One. 2020 Nov 24;15(11):e0232922. doi: 10.1371/journal.pone.0232922 (PMC7685467; doi:10.1371/journal.pone.0232922)
Supplement: S1 Table — (Np = pure deciduous forests, M = mixed deciduous-evergreen forests, Nb = pure evergreen forests) and landscapes (COA = coasts, MOU = mountains), showing: (i) species code, (ii) TAX = taxonomic group (Li = liverworts, Ms = mosses), (iii) GDP = global distribution patterns (D = disjunct with South America, South Africa and Europe; E = endemic; PAN = pantropical-type Podocarpus; A = austral-Antarctic; COS = cosmopolitan; B = bipolar), and (iv) substrates (LT = litter; DW = decaying wood; BS = bare soil; St = stones; EP = epiphytic on branches and bark in the forest floor). OF = occurrence frequency in each forest type and landscapes (%), and ẋ = mean frequency of occurrence in the entire study (%). (DOCX) [file pone.0232922.s003.docx]

**S1 Table. Bryophyte species observed in each forest type** (Np = pure deciduous forests, M = mixed deciduous-evergreen forests, Nb = pure evergreen forests) and landscapes (COA = coasts, MOU = mountains), showing: (i) species code, (ii) TAX = taxonomic group (Li = liverworts, Ms = mosses), (iii) GDP = global distribution patterns (D = disjunct with South America, South Africa and Europe; E = endemic; PAN = pantropical-type *Podocarpus*; A = austral-Antarctic; COS = cosmopolitan; B = bipolar), and (iv) substrates (LT = litter; DW = decaying wood; BS = bare soil; St = stones; EP = epiphytic on branches and bark in the forest floor). OF = occurrence frequency in each forest type and landscapes (%), and ẋ = mean frequency of occurrence in the entire study (%).

|  |  |  |  |  |  | **OF by Forest Type** | | | | **OF by Landscape** | | |
| --- | --- | --- | --- | --- | --- | --- | --- | --- | --- | --- | --- | --- |
| **Family** | **Species name** | **Code** | **TAX** | **GDP** | **Substrate** | **Np** | **M** | **Nb** | **ẋ** | **COA** | **MOU** | **ẋ** |
| Adelanthaceae | *Adelanthus lindbergianus* (Lehm.) Mitt. | ADLI | Li | D | LT-DW-EP | 25.00 | 15.00 | 55.00 | 31.67 | 30.00 | 33.30 | 31.65 |
| Lepidoziaceae | *Lepidozia chordulifera* Taylor | LECH | Li | E | LT-DW-BS-EP | -- | 25.00 | 30.00 | 27.50 | 13.30 | 23.30 | 18.30 |
| Lophocoleaceae | *Leptoscyphus huidobroanus* (Mont.) Gottsche | LEHU | Li | E | LT-DW-BS-EP | 15.00 | 10.00 | 20.00 | 15.00 | 30.00 | -- | 30.00 |
| Adelanthaceae | *Adelanthus integerrimus* Grolle | ADIN | Li | E | LT-DW-EP | -- | 20.00 | 10.00 | 15.00 | -- | 20.00 | 20.00 |
| Geocalycaceae | *Clasmatocolea* sp. | CLSP | Li | D | BS-EP | -- | 15.00 | -- | 15 | 6.70 | 3.30 | 5.00 |
| Lophocoleaceae | *Chiloscyphus hookeri* J.J. Engel | CHHO | Li | E | LT-DW-EP | -- | 5.00 | 20.00 | 12.5 | 6.70 | 10.00 | 8.35 |
| Lophocoleaceae | *Cryptolophocolea pallidovirens* (Hook. f. et Taylor) L.Söderstr. | CRPA | Li | E | LT-S | -- | -- | 10.00 | 10.00 | -- | 6.70 | 6.70 |
| Acrobolbaceae | *Tylimanthus renifolius* Hässel et Solari | TYRE | Li | E | LT-DW-EP | 5.00 | 15.00 | 5.00 | 8.33 | 3.30 | 13.30 | 8.30 |
| Geocalycaceae | *Leptoscyphus chiloscyphoideus* (Lehm.) Lindenb. | LECHI | Li | E | LT-EP | -- | 5.00 | 5.00 | 5.00 | 3.30 | 3.30 | 3.30 |
| Lophocoleaceae | *Lophocolea* sp. | LOPSP | Li | UN | LT-DW | -- | 5.00 | 5.00 | 5.00 | 3.30 | 3.30 | 3.30 |
| Geocalycaceae | *Heteroscyphus integrifolius (*Lehm et Lindenb) Fulf. | HEIN | Li | D | LT | 5.00 | -- | -- | 5.00 | -- | 3.30 | 3.30 |
| Lophoziaceae | *Lophozia* sp. | LOPS | Li | D | EP | -- | 5.00 | -- | 5.00 | 3.30 | -- | 3.30 |
| Dicranaceae | *Acrocladium auriculatum* (Mont.) Mitt. | ACAU | Ms | C | LT-DW-BS-EP | 70.00 | 60.00 | 15.00 | 48.33 | 33.30 | 63.30 | 48.30 |
| Dicranaceae | *Dicranoloma robustum* (Hook. f. & Wilson) Paris | DIRO | Ms | A | LT-DW-EP | 15.00 | 20.00 | 45.00 | 26.67 | 33.30 | 20.00 | 26.65 |
| Dicranaceae | *Dicranoloma chilense* (De Not.) Ochyra&Matteri | DICH | Ms | A | LT-DW-EP | 15.00 | 20.00 | 35.00 | 23.33 | 13.30 | 33.30 | 23.30 |
| Ditrichaceae | *Ditrichum cylindricarpum* (Müll. Hal.) F. Muell. | DICY | Ms | A | LT-DW-EP | -- | 30.00 | 35.00 | 32.50 | 10.00 | 33.30 | 21.65 |
| Dicranaceae | *Dicranoloma billardierii* (Brid.) Paris | DIBI | Ms | PAN | LT-DW-BS-EP | -- | 35.00 | 25.00 | 30.00 | 16.70 | 23.30 | 20.00 |
| Dicranaceae | *Campylopus clavatus* (R. Br.) Wilson | CACL | Ms | PAN | LT-DW-EP | -- | 30.00 | 10.00 | 20.00 | -- | 26.70 | 26.70 |
| Hypnaceae | *Hypnum skottsbergii* Ando. | HYSK | Ms | A | BS-S-EP | -- | 15.00 | -- | 15.00 | 10.00 | -- | 10.00 |
| Polytrichaceae | *Dendroligotrichum squamosum* (Hook. F. & Wilson) Broth. ex Cardot | DESQ | Ms | E | LT-DW-EP | 15.00 | 5.00 | -- | 10.00 | -- | 13.30 | 13.30 |
| Rhizogoniaceae | *Leptotheca gaudichaudii* Schwägr. | LEGA | Ms | A | LT-DW-BS-EP | -- | 10.00 | 10.00 | 10.00 | 13.30 | -- | 13.30 |
| Lepyrodontaceae | *Lepyrodon lagurus* (Hook.) Mitt. | LELA | Ms | A | LT-BS-EP | 10.00 | 10.00 | 5.00 | 8.33 | 16.70 | -- | 16.70 |
| Dicranaceae | *Dicranoloma* sp. | DISP | Ms | A | LT-EP | 5.00 | 5.00 | 10.00 | 6.67 | 6.70 | 6.70 | 6.70 |
| Bryaceae | *Pohlia nutans* (Hedw.) Lindb. | PONU | Ms | COS | LT-DW | 5.00 | 5.00 | 5.00 | 5.00 | 3.30 | 6.70 | 5.00 |
| Bartramiaceae | *Bartramia mossmaniana* Müll. Hal. | BAMO | Ms | B | LT | -- | 5.00 | -- | 5.00 | 3.30 | -- | 3.30 |
| Pottiaceae | *Hennediella densifolia* (Hook. f. & Wilson) R.H. Zander | HEDE | Ms | A | BS | 5.00 | -- | -- | 5.00 | 3.30 | -- | 3.30 |
| Rhizogoniaceae | *Hymenodontopsis mnioides* (Hook.) N.E. Bell. A.E. Newton & D. Quandt | HYMI | Ms | PAN | LT | -- | 5.00 | -- | 5.00 | 3.30 | -- | 3.30 |
