# Supplementary material for: Forest canopy-cover composition and landscape influence on bryophyte communities in Nothofagus forests of southern Patagonia
Source: PLoS One. 2020 Nov 24;15(11):e0232922. doi: 10.1371/journal.pone.0232922 (PMC7685467; doi:10.1371/journal.pone.0232922)
Supplement: S2 Table — BA = basal area (m2 ha-1), DH = dominant height (m), DBH = diameter at breast height (cm). (DOCX) [file pone.0232922.s004.docx]

**S2 Table. Generalized linear mixed models (GLMMs) to evaluate the effect of tree species contribution in the canopy composition in mixed deciduous (*N. pumilio*) and evergreen (*N. betuloides*) forests and landscapes (COA = coasts, MOU = mountains) over the forest structure variables.** BA = basal area (m^2^ ha^-1^), DH = dominant height (m), DBH = diameter at breast height (cm).

| **Factor** | | **BA** | **DH** | **DBH** |
| --- | --- | --- | --- | --- |
| Species | *N. pumilio* | 43.5 | 18.0 | 45.7 |
|  | *N. betuloides* | 33.6 | 18.4 | 53.5 |
|  | F | 2.37 | 0.06 | 2.82 |
|  | p | 0.132 | 0.803 | 0.102 |
| Landscapes | COA | 32.7 | 17.2 | 54.5 b |
|  | MOU | 44.4 | 19.2 | 44.7 a |
|  | F | 3.32 | 1.15 | 4.44 |
|  | p | 0.077 | 0.292 | 0.042 |
| Interaction | F | 1.36 | 0.04 | 4.09 |
|  | p | 0.251 | 0.844 | 0.051 |

F, p = F test, probability. Different letters in each column show significant differences based on the LSD Fisher’s test at p < 0.05.
