# Supplementary material for: Forest canopy-cover composition and landscape influence on bryophyte communities in Nothofagus forests of southern Patagonia
Source: PLoS One. 2020 Nov 24;15(11):e0232922. doi: 10.1371/journal.pone.0232922 (PMC7685467; doi:10.1371/journal.pone.0232922)
Supplement: S3 Table — The estimated sample coverage for a sample size 500 and 1000 (sample size Observed = 500 and Expected by extrapolation = 1000) were presented between brackets. Pure deciduous N. pumilio forests in the coasts (CNp) and mountains (MNp), pure evergreen N. betuloides forests in the coasts (CNb) and mountains (MNb), mixed deciduous-evergreen forests in the coasts (CM) and mountains (CM). (DOCX) [file pone.0232922.s005.docx]

**S3 Table. Rarefaction and extrapolation data through the estimated diversity by Hill number (q = 0, 1, 2) of order q for a sample size 500 and 1000.** The estimated sample coverage for a sample of size 500 and 1000 (sample size Observed = 500 and Expected by extrapolation = 1000) were presented between brackets. Pure deciduous *N. pumilio* forests in the coasts (CNp) and mountains (MNp), pure evergreen *N. betuloides* forests in the coasts (CNb) and mountains (MNb), mixed deciduous- evergreen forests in the coasts (CM) and mountains (CM).

| **Liverworts** | **q = 0** | | **q = 1** | | **q = 2** | |
| --- | --- | --- | --- | --- | --- | --- |
|  | **Observed = 500** | **Expected = 1000** | **Observed = 500** | **Expected = 1000** | **Observed = 500** | **Expected = 1000** |
| **CNp** | 2.0 (100 %) | 2.0 (100%) | 1.9 (89%) | 2.0 (98%) | 1.8 (98%) | 2.0 (100%) |
| **CM** | 5.0 (63 %) | 7.2 (81 %) | 4.5 (63%) | 6.2 (81 %) | 4.0 (63%) | 5.3 (81%) |
| **CNb** | 7.0 (85%) | 9.9 (92 %) | 4.8 (85 %) | 5.6 (92%) | 4.1 (85%) | 4.4 (92%) |
| **MNp** | 3.0 (67 %) | 3.8 (95%) | 2.4 (67 %) | 3.1 (95%) | 2.0 (67%) | 2.4 (95%) |
| **MM** | 7.0 (90 %) | 8.9 (96%) | 5.3 (90%) | 6.0 (96%) | 4.8 (90%) | 5.2 (96%) |
| **MNb** | 9.0 (94%) | 11.2 (97%) | 6.2 (94%) | 6.7 (97%) | 5.3 (94%) | 5.6 (97%) |
| **Mosses** | **q = 0** | | **q = 1** | | **q = 2** | |
|  | **Observed = 500** | **Expected = 1000** | **Observed = 500** | **Expected = 1000** | **Observed = 500** | **Expected = 1000** |
| **CNp** | 5.0 (86%) | 7.2 (93%) | 2.4 (86%) | 2.7 (93%) | 1.7 (86%) | 1.8 (93%) |
| **CM** | 11.0 (92%) | 12.3 (99%) | 8.2 (92%) | 9.2 (99%) | 6.4 (92%) | 7.0 (99%) |
| **CNb** | 7.0 (88%) | 8.7 (97%) | 3.9 (88%) | 4.4 (97%) | 2.6 (88%) | 2.7 (97%) |
| **MNp** | 6.0 (97%) | 6.4 (100%) | 4.3 (98%) | 4.6 (100%) | 3.4 (97%) | 3.6 (100%) |
| **MM** | 8.0 (98 %) | 8.2 (100%) | 5.3 (97%) | 5.5 (100%) | 4.2 (98%) | 4.3 (100%) |
| **MNb** | 7.0 (99%) | 7.4 (100%) | 3.4 (99%) | 3.5 (100%) | 2.3 (99%) | 2.4 (100%) |
