# Supplementary material for: Forest canopy-cover composition and landscape influence on bryophyte communities in Nothofagus forests of southern Patagonia
Source: PLoS One. 2020 Nov 24;15(11):e0232922. doi: 10.1371/journal.pone.0232922 (PMC7685467; doi:10.1371/journal.pone.0232922)
Supplement: S4 Table — BA = basal area (m2 ha-1), DH = dominant height (m), DBH = diameter at breast height (cm), CC = canopy cover (%), RLAI = relative leaf area index, TR = transmitted total solar radiation (%), SM = soil moisture (%), PP = effective annual precipitation (mm yr-1), ST = soil temperature (°C), AT = air temperature (°C), RH = relative air humidity (%), S = slope (%), pH, R = resistance to penetration (N cm-2), BS = bare soil cover (%), Ds = debris cover (%), VC = vascular plant cover including ferns, monocots and dicots (%), and L = lichen cover (%). Correlation coefficients varied between -1 to +1. * = showed correlations values over 0.5 and under -0.5, with p-values < 0.05. (DOCX) [file pone.0232922.s006.docx]

**S4 Table. Pearson correlation coefficients obtained between the tested variables of the Canonical Correspondence Analysis.** BA = basal area (m^2^ ha^-1^), DH = dominant height (m), DBH = diameter at breast height (cm), CC = canopy cover (%), RLAI = relative leaf area index, TR = transmitted total solar radiation (%), SM = soil moisture (%), PP = effective annual precipitation (mm yr^-1^), ST = soil temperature (°C), AT = air temperature (°C), RH = relative air humidity (%), S = slope (%), pH, R = resistance to penetration (N cm^-2^), BS = bare soil cover (%), Ds = debris cover (%), VC = vascular plant cover including ferns, monocots and dicots (%), and L = lichen cover (%).

|  | **BA** | **DH** | **DBH** | **CC** | **RLAI** | **TR** | **SM** | **PP** | **ST** | **AT** | **RH** | **S** | **pH** | **R** | **BS** | **Ds** | **CV** | **L** |
| --- | --- | --- | --- | --- | --- | --- | --- | --- | --- | --- | --- | --- | --- | --- | --- | --- | --- | --- |
| **BA** | 1 |  |  |  |  |  |  |  |  |  |  |  |  |  |  |  |  |  |
| **DH** | -0.284 | 1 |  |  |  |  |  |  |  |  |  |  |  |  |  |  |  |  |
| **DBH** | **-0.524*** | **0.637*** | 1 |  |  |  |  |  |  |  |  |  |  |  |  |  |  |  |
| **CC** | 0.189 | 0.010 | -0.173 | 1 |  |  |  |  |  |  |  |  |  |  |  |  |  |  |
| **RLAI** | 0.248 | -0.045 | -0.191 | **0.957*** | 1 |  |  |  |  |  |  |  |  |  |  |  |  |  |
| **TR** | -0.147 | -0.022 | 0.137 | **-0.967*** | **-0.890*** | 1 |  |  |  |  |  |  |  |  |  |  |  |  |
| **VSW** | 0.301 | -0.078 | -0.157 | 0.099 | 0.111 | -0.113 | 1 |  |  |  |  |  |  |  |  |  |  |  |
| **PP** | -0.437 | 0.298 | 0.274 | -0.211 | -0.301 | 0.156 | -0.494 | 1 |  |  |  |  |  |  |  |  |  |  |
| **ST** | -0.221 | 0.231 | 0.268 | -0.003 | -0.062 | 0.028 | -0.232 | 0.070 | 1 |  |  |  |  |  |  |  |  |  |
| **AT** | -0.052 | -0.221 | -0.170 | 0.001 | 0.028 | 0.053 | **-0.637*** | -0.004 | 0.265 | 1 |  |  |  |  |  |  |  |  |
| **RH** | -0.094 | -0.239 | -0.255 | -0.094 | -0.104 | 0.101 | **-0.630*** | 0.434 | -0.233 | **0.741*** | 1 |  |  |  |  |  |  |  |
| **S** | 0.190 | -0.116 | -0.246 | -0.145 | -0.186 | 0.090 | 0.078 | -0.016 | -0.282 | 0.027 | 0.209 | 1 |  |  |  |  |  |  |
| **pH** | -0.366 | **0.618*** | **0.596*** | -0.030 | 0.023 | 0.032 | -0.204 | 0.181 | 0.232 | -0.061 | -0.194 | -0.357 | 1 |  |  |  |  |  |
| **R** | -0.352 | **0.533*** | **0.454** | -0.031 | -0.015 | 0.066 | **-0.621*** | 0.232 | 0.361 | **0.571*** | 0.340 | -0.147 | 0.465 | 1 |  |  |  |  |
| **BS** | 0.115 | -0.197 | -0.184 | 0.032 | 0.050 | -0.003 | -0.306 | -0.014 | -0.350 | **0.510*** | **0.631*** | 0.367 | 0.226 | 0.226 | 1 |  |  |  |
| **Ds** | 0.024 | 0.201 | 0.016 | 0.134 | 0.006 | -0.128 | -0.112 | -0.125 | -0.041 | 0.221 | 0.151 | 0.115 | 0.254 | 0.217 | 0.335 | 1 |  |  |
| **CV** | -0.286 | 0.445 | 0.472 | -0.007 | 0.101 | -0.001 | 0.034 | 0.365 | 0.398 | -0.418 | -0.421 | 0.019 | 0.468 | 0.024 | -0.491 | 0.015 | 1 |  |
| **L** | -0.156 | -0.077 | -0.065 | -0.025 | -0.073 | 0.021 | -0.375 | 0.366 | -0.123 | 0.201 | 0.432 | 0.145 | 0.104 | 0.031 | 0.280 | 0.192 | 0.081 | 1 |

Correlation coefficients varied between -1 to +1. * = showed correlations values over 0.5 and under -0.5, with p-values < 0.05.
