# Supplementary material for: Forest canopy-cover composition and landscape influence on bryophyte communities in Nothofagus forests of southern Patagonia
Source: PLoS One. 2020 Nov 24;15(11):e0232922. doi: 10.1371/journal.pone.0232922 (PMC7685467; doi:10.1371/journal.pone.0232922)
Supplement: S5 Table — LT = litter, DW = decaying wood, BS = bare soil, St = stones, EP = epiphyte of branches and bark in the forest floor. Pure deciduous N. pumilio forests in the coasts (CNp) and mountains (MNp), pure evergreen N. betuloides forests in the coasts (CNb) and mountains (MNb), mixed deciduous-evergreen forests in the coasts (CM) and mountains (CM). (DOCX) [file pone.0232922.s007.docx]

**S5 Table. Crosstabs of frequency and chi-square test of liverworts and mosses for each substrate.** LT = litter, DW = decaying woods, BS = bare soil, St = stones, EP = epiphyte of branches and bark in the forest floor. Pure deciduous *N. pumilio* forests in the coasts (CNp) and mountains (MNp), pure evergreen *N. betuloides* forests in the coasts (CNb) and mountains (MNb), mixed deciduous-evergreen forests in the coasts (CM) and mountains (CM).

| Liverworts = Chi-square (Pearson) = 22.90; df = 20; p = 0.289 |  | **LT** | **DW** | **BS** | **St** | **EP** | **Total** |
| --- | --- | --- | --- | --- | --- | --- | --- |
| **CNp** | Frequency | 2.0 | 2.0 | 2.0 | 0.0 | 2.0 | 8.0 |
|  | ExpectedFrequency | 3.7 | 1.8 | 0.5 | 0.1 | 1.8 | 8.0 |
| **CM** | Frequency | 2.0 | 2.0 | 2.0 | 0.0 | 2.0 | 8.0 |
|  | ExpectedFrequency | 3.7 | 1.8 | 0.5 | 0.1 | 1.8 | 8.0 |
| **CNb** | Frequency | 10 | 3.0 | 1.0 | 0.0 | 3.0 | 17.0 |
|  | ExpectedFrequency | 7.8 | 3.9 | 1.1 | 0.2 | 3.9 | 17.0 |
| **MNp** | Frequency | 4.0 | 0.0 | 0.0 | 0.0 | 0.0 | 4.0 |
|  | ExpectedFrequency | 1.8 | 0.9 | 0.3 | 0.1 | 0.9 | 4.0 |
| **MM** | Frequency | 8.0 | 5.0 | 0.0 | 0.0 | 7.0 | 20.0 |
|  | ExpectedFrequency | 9.2 | 4.6 | 1.4 | 0.3 | 4.6 | 20.0 |
| **MNb** | Frequency | 8.0 | 5.0 | 0.0 | 1.0 | 3.0 | 17.0 |
|  | ExpectedFrequency | 7.8 | 3.9 | 1.1 | 0.2 | 3.9 | 17.0 |
| **Total** | Frequency | 34.0 | 17.0 | 5.0 | 1.0 | 17.0 | 74.0 |
|  | ExpectedFrequency | 34.0 | 17.0 | 5.0 | 1.0 | 17.0 | 74.0 |
| Mosses = Chi-square (Pearson) = 77.08; df = 25; p < 0.001 |  | **LT** | **DW** | **BS** | **S** | **EP** | **Total** |
| **CNp** | Frequency | 2.0 | 5.0 | 3.0 | 0.0 | 7.0 | 17.0 |
|  | ExpectedFrequency | 8.8 | 2.9 | 1.1 | 0.1 | 4.0 | 17.0 |
| **CM** | Frequency | 12.0 | 2.0 | 6.0 | 1.0 | 10.0 | 31.0 |
|  | ExpectedFrequency | 16.1 | 5.4 | 2.1 | 0.2 | 7.2 | 31.0 |
| **CNb** | Frequency | 13.0 | 1.0 | 0.0 | 0.0 | 2.0 | 16.0 |
|  | ExpectedFrequency | 8.3 | 2.8 | 1.1 | 0.1 | 3.7 | 16.0 |
| **MNp** | Frequency | 13.0 | 2.0 | 0.0 | 0.0 | 5.0 | 20.0 |
|  | ExpectedFrequency | 10.4 | 3.5 | 1.3 | 0.1 | 4.7 | 20.0 |
| **MM** | Frequency | 17.0 | 9.0 | 1.0 | 0.0 | 7.0 | 34.0 |
|  | ExpectedFrequency | 17.7 | 5.9 | 2.3 | 0.2 | 7.9 | 34.0 |
| **MNb** | Frequency | 21.0 | 7.0 | 0.0 | 0.0 | 4.0 | 32.0 |
|  | ExpectedFrequency | 16.6 | 5.5 | 2.1 | 0.2 | 7.5 | 32.0 |
| **Total** | Frequency | 78.0 | 26.0 | 10.0 | 1.0 | 35.0 | 150.0 |
|  | ExpectedFrequency | 78.0 | 26.0 | 10.0 | 1.0 | 35.0 | 150.0 |
